# CenIR, an essential BlaIR-family regulatory system in *C. difficile*

**DOI:** 10.1128/jb.00196-26

**Published:** 2026-07-14

**Authors:** Micaila P. Kurtz, Ute Müh, David S. Weiss, Craig D. Ellermeier

**Affiliations:** 1 Department of Microbiology and Immunology, Carver College of Medicine, University of Iowa 311821, Iowa City, Iowa, USA; 2 Graduate Program in Genetics, University of Iowa 4083, Iowa City, Iowa, USA; The Ohio State University, Columbus, Ohio, USA

**Keywords:** cell envelope, antibiotic resistance, signal transduction, gene regulation

## Abstract

The CenIR regulatory system of *Clostridioides difficile* comprises a predicted transcriptional repressor, CenI, and a predicted membrane metalloprotease, CenR. The physiological role of CenIR and activating signal(s) are not known. CenIR belongs to the BlaIR family of regulators that mediate resistance to β-lactam antibiotics. In canonical BlaIR systems, binding of a β-lactam to the extracellular transpeptidase domain of BlaR triggers proteolysis of BlaI and, thus, induction of a closely linked β-lactamase gene. However, CenR lacks a β-lactam-binding domain and transposon mutagenesis indicated CenI is essential for viability even when β-lactams are not present. Here, we confirmed the essentiality of CenIR and determined its regulon contains ~12 genes, including an exported protein of unknown function (CDR_0474) that is induced about 500-fold and a peptidoglycan hydrolase (Cwp6) that is induced about 7-fold when cells are depleted of CenIR. There are no essential genes or β-lactamases in the regulon. Phenotypic characterization of CenIR-depletion strains revealed slower growth, mild elongation, and cell lysis. Deletion of *cdr_0474* corrected all three defects, while deletion of *cwp6* only rescued the lysis phenotype. It was possible to delete *cenIR* in either a Δ*cdr_0474* or Δ*cwp6* background. We propose that CenIR is essential because its absence leads to lysis due to Cwp6 overproduction. Bioinformatic analyses revealed the predicted extracellular sensing domains in annotated “BlaR” proteins are diverse. Thus, BlaIR systems are not dedicated to defense against β-lactams but probably enable bacteria to adapt to a variety of environmental stimuli.

**IMPORTANCE:**

Many of the regulatory systems for controlling cell envelope biogenesis and stress responses have yet to be studied. Here, we characterize a *Clostridioides difficile* BlaIR-like regulatory system that we have named CenIR for cell envelope. Unlike canonical BlaIR systems, which bind β-lactams and induce a β-lactamase, CenIR lacks a β-lactam binding domain and is essential for viability even in the absence of antibiotics. We identified the genes in the regulon and found that CenIR is essential because its absence leads to the overproduction of the Cwp6 peptidoglycan hydrolase. We also show that most annotated BlaIR-like systems lack a β-lactam-binding domain, from which we infer that these systems have much broader physiological roles than generally appreciated.

## INTRODUCTION

*Clostridioides difficile* is a Gram-positive, spore-forming gastrointestinal pathogen that causes symptoms ranging from mild diarrhea to lethal pseudomembraneous colitis (1). *C. difficile* resides in a complex environment and encodes a large number of regulatory circuits, including roughly 50 two-component signaling systems (2). There are also seven genes annotated as BlaR-like, four of which are adjacent to a BlaI homolog. BlaIR systems are widespread in Gram-positive bacteria and have been especially well-studied in *Bacillus lichenoformis* and *Staphylococcus aureus*, where the system is known as Mec (3). In these systems, BlaI is a transcriptional repressor and BlaR is a membrane metalloprotease with an extracellular penicillin-binding transpeptidase (TP) domain (PF00905). Binding of a β-lactam to the TP domain turns on BlaR’s latent protease activity, which resides in a conserved M56 peptidase domain (PF05569). Activated BlaR cleaves BlaI to induce the target β-lactamase. Only one of *C. difficile’s* BlaIR-like systems has been characterized; it responds to β-lactams like a canonical BlaIR system (4).

BlaIR systems are not essential in any organism where they have been studied, nor would essentiality be expected in the absence of β-lactams. We were therefore intrigued when a gene pair annotated as “*blaIR*-like” was identified as essential for vegetative growth in a large-scale transposon mutagenesis of *C. difficile* strain R20291 (5). The genes in question are the *blaI* homolog *cdr_0472* and the *blaR* homolog *cdr_0473*. (Note that locus names in this report have been shortened from *cdr20291_xxxx* to *cdr_xxxx.*) Close inspection of the BlaR homolog suggests it does not sense β-lactams because it lacks a predicted TP domain. Moreover, essentiality under standard laboratory growth conditions argues for a role other than (or at least beyond) mounting a stress response. Thus, *cdr_0472* and *cdr_0473* appeared to encode an important but non-canonical BlaIR-like system.

Here, we confirm the essentiality of this gene pair, which we have named *cenI* (*cdr_0472*) and *cenR* (*cdr_0473*) for cell envelope. Depleting *C. difficile* of CenIR induced expression of multiple cell envelope genes, resulting in slower growth, modest elongation, and eventual lysis. Slower growth and elongation were traced to induction of an exported protein of unknown function, while the lysis phenotype was traced to induction of a cell wall hydrolase, Cwp6. We were unable to identify the inducing signal, despite some effort. Nevertheless, multiple lines of evidence argue it is not β-lactams. Interestingly, bioinformatic analyses indicate that fewer than 10% of annotated “BlaR” proteins have a TP domain. Thus, non-canonical BlaIR systems like CenIR appear to be the rule rather than the exception.

## RESULTS

**Fig 1:**
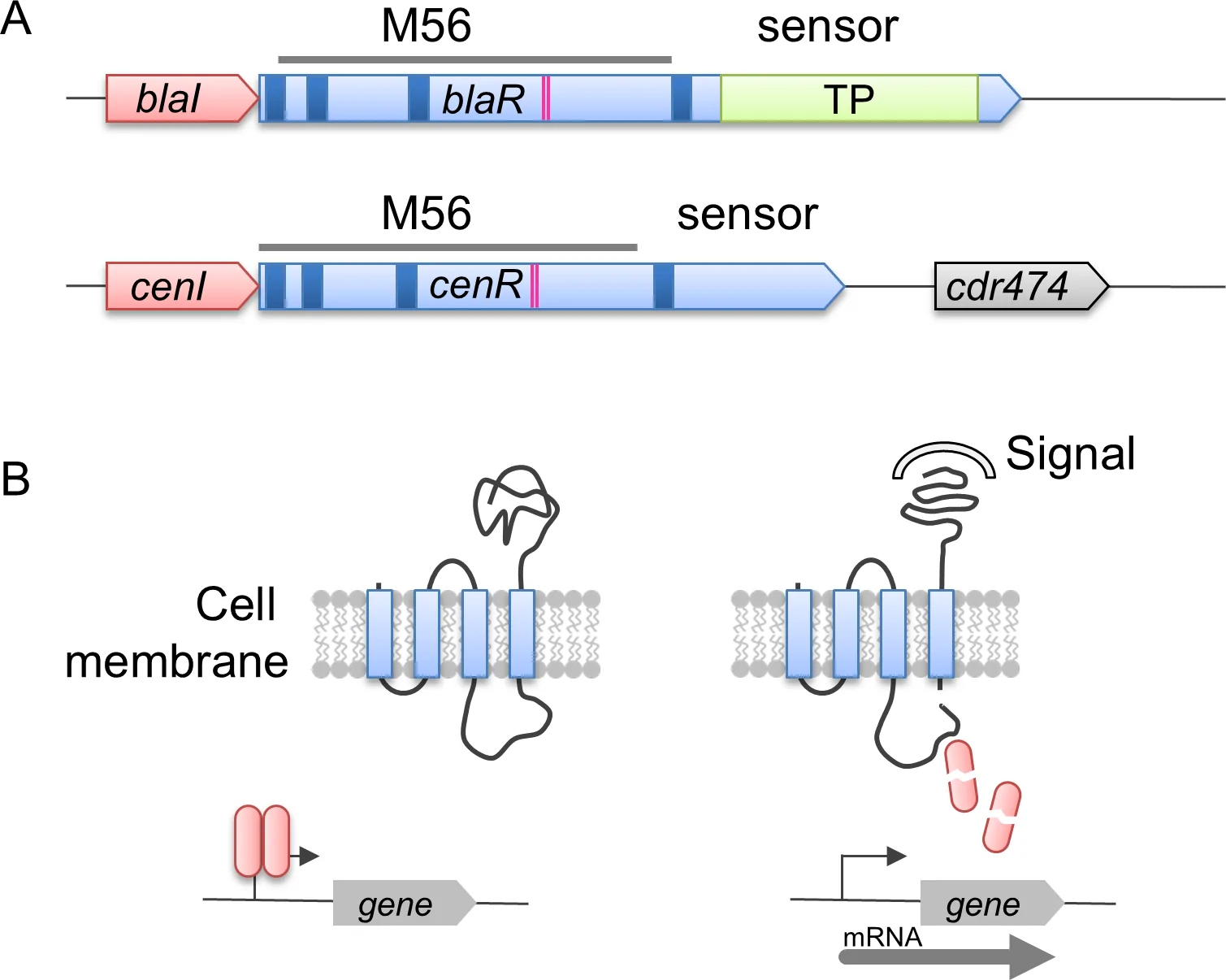
Model of CenIR operon and regulation. (**A**) Operon structure of *cenIR* and comparison to *blaIR*. Genes *cenI* and *blaI* (red) encode a transcriptional repressor that binds DNA as a dimer. Genes *cenR* and *blaR* (blue) encode a sensor protease with an N-terminal M56 peptidase domain that includes four transmembrane helices (dark blue) and zinc-binding histidines (magenta). The C-terminal end of BlaR has a transpeptidase domain (TP, green) that binds β-lactams. In contrast, the C-terminal part of CenR is about 100 amino acids shorter and not homologous to any known domain. AlphaFold modeling predicts the C-terminus of CenR to be largely disordered. (**B**) Model of regulation based on analogy to BlaR. CenR’s disordered domain binds to an unknown signal, inducing a conformational change that activates autoproteolysis near the 4th TM, followed by cleavage of CenI, and induction of the regulon.

The genes *cenI and cenR* are predicted to be in a two-gene operon with one transcriptional start site (as described for strain 630 [6]; Fig. 1A). Like canonical BlaI proteins, CenI has a predicted helix-turn-helix DNA-binding motif (3). CenR, like canonical BlaR proteins, is predicted to have a cytoplasmic N-terminal Peptidase_M56 domain that is anchored in the membrane through four transmembrane helices. However, the C-terminal TP domain characteristic of canonical BlaR proteins is replaced in CenR with a shorter domain (by ~100 amino acids) that is predicted to be largely disordered (Fig. 1B). Thus, the predicted structures of CenI and CenR suggest CenI binds as a dimer to target genes to repress their expression. Interaction of something other than a β-lactam with CenR’s extracellular sensing domain triggers proteolysis of CenI and gene induction.

### CenIR-depletion slows growth, increases cell length, and promotes lysis

**Fig 2:**
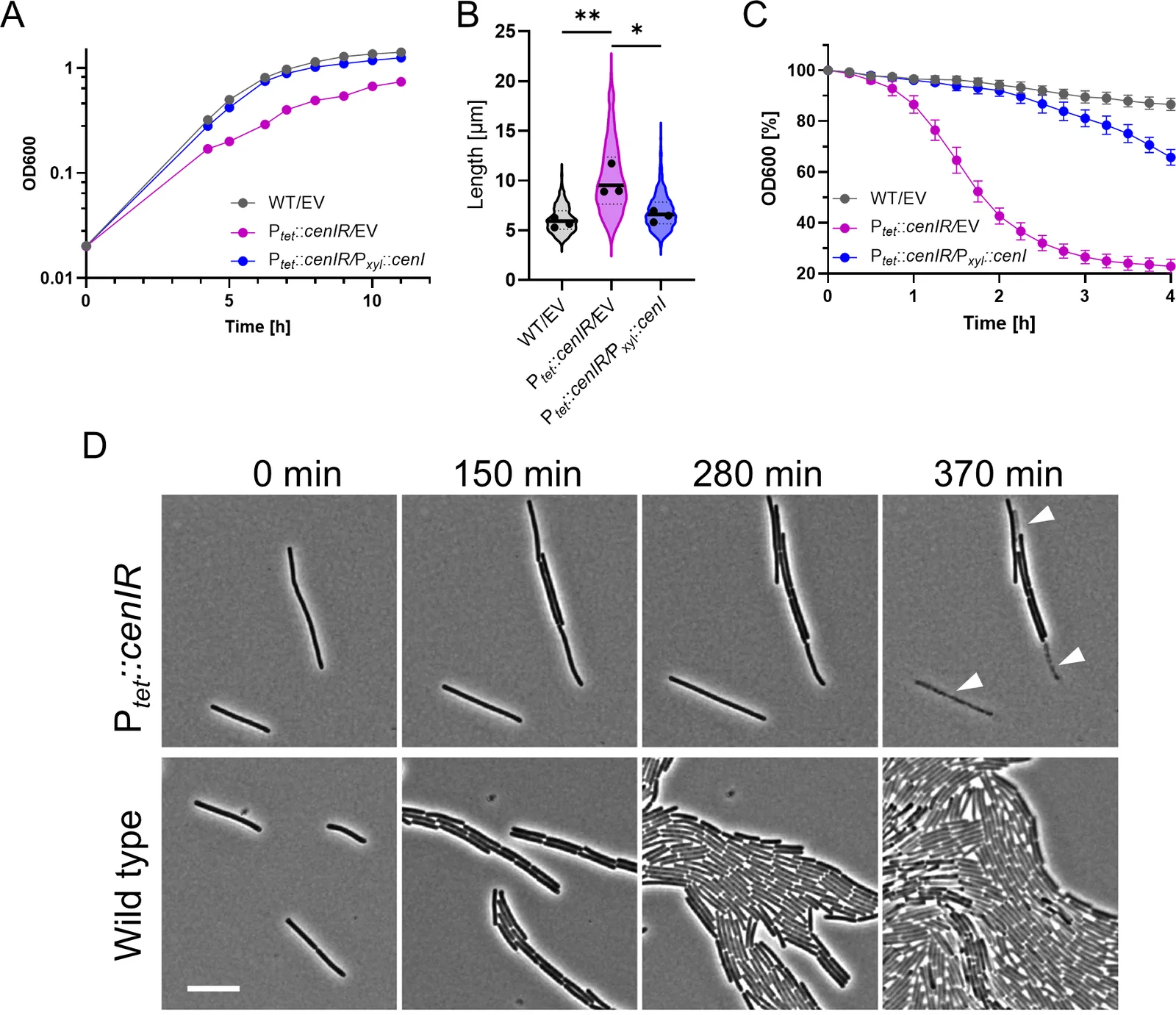
Depletion of *cenIR* causes slower growth, elongation, and increased lysis. (**A**) Growth curves. Cultures that had been grown overnight in TY-thi with 2 ng/mL aTet were subcultured 1:50 into TY-thi with 1% xylose. At ~5 h, cultures were sampled for phase contrast microscopy to determine cell length (**B**). At 6 h, the wild type and the complemented strain were back-diluted fivefold such that all strains would reach an OD_600_ ~ 0.7 at 11 h when cells were harvested for the lysis assay (**C**). Experiments were performed on three different days. Growth curves are representative. Cell lengths are graphed as violin plots using pooled data from all three experiments (≥200 cells per experiment). Bars and dotted lines are the mean and quartiles, respectively. Dots are the means of each experiment. Lysis is plotted as the mean ± SD from all three experiments. Statistical analysis was by one-way ANOVA (*n* = 3, *P ≤ 0.05, **P ≤ 0.01). (**D**) Select images from time-lapse microscopy of the CenIR depletion strain and wild type, both grown in the absence of aTet. Note that the depletion strain grew slower, elongated, and eventually lysed (white carets). Size bar: 10 µm. Strains: WT/EV (UM1173), P*_tet_::cenIR/*P*_tet_::cenIR* (MK212), P*_tet_::cenIR*/EV (MK230), P*_tet_::cenIR* (UM1364).

Initially, we attempted to delete *cenIR* using CRISPR mutagenesis but were unsuccessful. The same CRISPR editing plasmid was later used to delete *cenIR* in different strain backgrounds (described below), suggesting the inability to delete *cenIR* in wild type is due to biological rather than technical reasons. Instead of deleting *cenIR*, we constructed a depletion strain by using CRISPR mutagenesis to insert a P*_tet_* promoter in front of the *cenIR* operon. The P*_tet_::cenIR* depletion strain grew well in the presence of the inducer anhydrotetracycline (aTet), but growth slowed upon transfer to media lacking aTet (Fig. 2A; Fig. S1A). Depleted cells were longer than WT and lysed faster in an *in vitro* assay in which cells are suspended in phosphate buffer containing 0.01% Triton X-100 (Fig. 2B and C). Note that the rate of lysis is dependent on the growth phase when cells are harvested (Fig. S2); care was therefore taken to harvest cells at the same OD_600_. The OD_600_ at time of harvest ranged from 0.6 to 0.8 in the various experiments throughout this report but was always carefully matched within an experiment. All phenotypic changes were restored to near wild type when *cenI* was expressed from a plasmid under P*_xyl_* control (Fig. 2A through C; Fig. S1A). Elongation and lysis were also observed in time-lapse microscopy of live cells of the P*_tet_::cenIR* strain growing under depletion conditions on an agarose pad (Fig. 2D; Videos S1 to S4). Curiously, however, in spot titer assays in the absence of aTet, the depletion strain grew out to the same dilution as a WT control (Fig. S3A). We had expected a more profound viability defect in view of our inability to delete *cenIR*.

The phenotypic consequences of CenIR-depletion were also investigated using CRISPR interference (CRISPRi [7]) against *cenIR*. Although viability in a spot titer assay was unaffected, cells scraped from the plates were elongated and showed signs of lysis (Fig. S3B). Thus, P_tet_-based depletion or CRISPRi knockdown are in agreement—we observed similar morphological changes but no drop in viability.

Viability in the spot titer assay is difficult to reconcile with our inability to delete *cenIR*. We considered that residual *cenIR* expression under depletion conditions might ameliorate phenotypic defects, but using CRISPRi to (further) silence expression of P*_tet_::cenIR* in the absence of aTet had no effect on viability in a spot titer assay. Importantly, CenIR-depletion caused lysis in multiple assay formats (lysis assay, spot titer, time-lapse microscopy), and as will be shown below, we were eventually able to delete *cenIR* once we identified and removed the genes responsible for lysis. Thus, we consider *cenIR* to be essential or quasi-essential. A similar discrepancy emerged from large-scale Tn mutagenesis, where *cenIR* was classified as essential in an early study (5) but not in a more recent one (8).

### The CenIR regulon includes cell envelope genes

We speculated that identifying the genes in the CenIR regulon would shed light on its physiological function. To this end, we determined transcriptional profiles with and without CRISPRi knockdown of *cenIR*. CRISPRi was chosen over the P*_tet_*-based depletion strain because pilot studies showed that the high aTet required to fully phenocopy WT inhibits growth. Note that we use the term regulon loosely here and include genes that respond directly or indirectly to CRISPRi against *cenIR*.

Strains of *C. difficile* R20291 harboring either a CRISPRi plasmid targeting *cenI* or a negative control plasmid that expresses a non-targeting sgRNA were grown under CRISPRi-inducing conditions (i.e., 1% xylose). Cultures were maintained by repeated back-dilution for roughly 12 doublings, long enough to see a mild effect on growth rate (Fig. S3C). Cells were processed for RNA-seq as described previously (9).

RNA-seq analysis identified 14 genes whose transcript abundance changed by ≥4-fold (all *P*-values were ≤10^−8^) upon CRISPRi silencing of *cenIR* (Table 1; Table S1). Only two of these genes were repressed, *cenI* and *cenR*, which were down four- and sevenfold, respectively. Repression of these genes demonstrates that CRISPRi worked as intended and was specific for *cenI* (which is polar onto *cenR*) without any strong off-target effects. Consistent with CenI being a repressor, all other affected genes exhibited increased transcript levels, mostly in the range of four- to eightfold. A remarkable exception is *cdr_0474*, which is located 215 bp downstream of *cenIR* (Fig. 1) and for which mRNA abundance increased by 500-fold. CDR_0474 is a small protein of 136 amino acids found exclusively in *C. difficile*. It is predicted to have an N-terminal signal sequence, implying it is exported. Consistent with this, CDR_0474 has been detected in the secreted proteome (10). BLAST searches failed to identify similarity to any other proteins.

**TABLE 1:** Genes with fourfold change or more in transcript levels upon CRISPRi silencing *cenIR*

Locus	Gene	Annotation	Log_2_ fold change	*P* adjusted
*cdr_0472*	*cenI*	*blaI*-like	−2.06	2.7E−08
*cdr_0473*	*cenR*	*blaR*-like	−2.82	2.0E−21
*cdr_0474*		Putative exported protein	9.11	0.0E+00
*cdr_2121*	*sinR*	HTH-type transcriptional regulator	2.99	3.5E−20
*cdr_2122*	*sinR'*	HTH-type transcriptional regulator	2.72	6.6E−18
*cdr_2227*	*macA*	4-Hydroxybutyrate dehydrogenase	2.15	3.6E−11
*cdr_2230*	*abfD*	Gamma-aminobutyrate metabolism dehydratase/isomerase	2.04	6.7E−09
*cdr_2231*	*sucD*	Succinate-semialdehyde dehydrogenase	2.06	1.5E−08
*cdr_2232*	*cat1*	Succinyl-CoA:coenzyme A transferase	2.16	1.2E−09
*cdr_2233*	*yidE*	Putative succinate transporter	2.29	6.5E−12
*cdr_2238*	*grdC*	Glycine reductase complex component C subunit beta	2.01	2.4E−10
*cdr_2241*	*grdE*	Glycine reductase complex component B subunits alpha and beta	2.09	7.9E−09
*cdr_2672*	*cwp6*	N-acetylmuramoyl-L-alanine amidase	2.90	3.6E−25
*cdr_2797*	*ldt1*	L,D-transpeptidase 1	2.68	1.5E−22

Genes that were derepressed at more moderate levels fall into three categories—regulation, metabolism, and peptidoglycan (PG) modification. (i) SinR and SinR’ are regulatory proteins that in *C. difficile* have pleiotropic effects on sporulation, toxin production, and motility (11). (ii) Genes *cdr_2227* through *cdr_2241* are part of a large gene cluster involved in fermentation of succinate and glycine. All additional genes in this cluster were also induced although by less than the fourfold cutoff for inclusion in Table 1 (Table S1). (iii) Lastly, *cdr_2672* codes for Cwp6, a PG hydrolase (*N*-acetylmuramoyl-L-alanine amidase) and *cdr_2797* codes for Ldt1, a L,D-transpeptidase that makes 3-3 crosslinks in PG. In view of the morphological defects we observed upon CenIR depletion, we chose to follow-up on the cell envelope-associated genes *cdr_0474, cwp6* and *ldt1*.

### Ldt1 is not linked to any of the morphological defects

**Fig 3:**
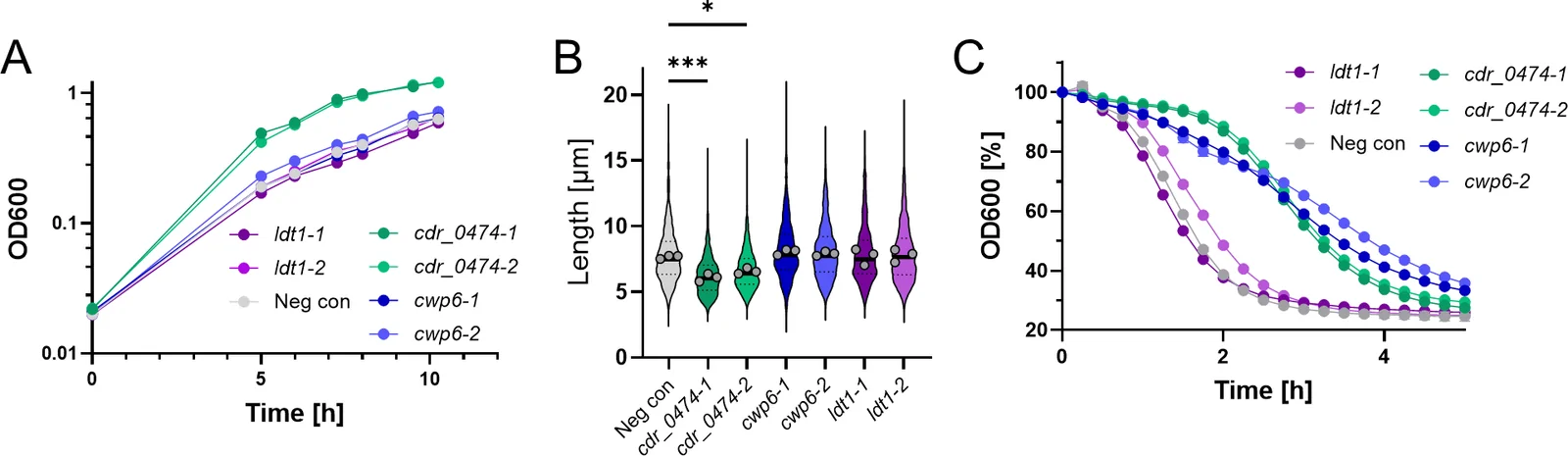
Effect of CRISPRi silencing of select regulon members on CenIR depletion phenotypes. (**A**) Growth curves. Strains grown overnight in TY-thi with 2 ng/mL aTet were subcultured 1:50 into TY-thi with 1% xylose and grown for about 10 h. At 8 h, cultures were sampled for microscopy to determine cell length (**B**). The two faster growing strains were back diluted sevenfold at 6 h to ensure that all strains were at an OD_600_ ~ 0.65 at 10.5 h when cells were harvested for the lysis assay (**C**). Growth curves and lysis assay are representative of three experiments. Cell lengths are graphed as violin plots using pooled data from all three experiments (≥200 cells per experiment). The bar and dotted lines are the mean and quartiles. Dots are the mean of each experiment. Statistical analysis is by one-way ANOVA, *n* = 3 (*P ≤ 0.05, ***P ≤ 0.001). The strains shown are the P*_tet_::cenIR* depletion strain containing various CRISPRi plasmids with sgRNAs targeting the indicated gene. “Neg con” is a control with an sgRNA that does not target anywhere in the genome. The strains are: “Neg con” (MK231), *ldt1-1* (MK227), *ldt1-2* (MK228), *cdr_474-1* (MK216), *cdr_474-2* (MK229), *cwp6-1* (MK217), *cwp6-2* (MK218).

We used CRISPRi to silence expression of *ldt1* in the P*_tet_::cenIR* depletion background. None of the morphological defects associated with CenIR depletion were affected (Fig. 3; Fig. S1B). We have shown elsewhere that one of the CRISPRi plasmids used in this experiment is effective at reducing Ldt1 levels, so the lack of phenotypic changes is not due to ineffective guides (12). This outcome is not too surprising because *C. difficile* has five *ldt* genes, of which three (including *ldt1*) show considerable functional redundancy and are alone sufficient for wild-type growth rate and morphology (12). Based on these considerations, we decided not to pursue Ldt1 further by constructing a deletion mutant. We did, however, use anti-Ldt1 anti-serum to examine Ldt1 abundance by Western blotting. Depletion of CenIR increased Ldt1 levels about twofold (Fig. S4), less than the fourfold increase indicated by RNA-seq but still in the same direction. In summary, Ldt1 is, indeed, elevated upon CenIR depletion, but this increase does not appear to be connected to any phenotypic changes.

### Deletion of *cdr_0474* corrects phenotypic defects caused by loss of CenIR

**Fig 4:**
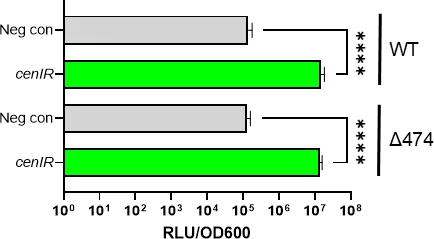
Depletion of CenIR induces P*_cdr_0474_::sluc^opt^*. The P*_cdr_0474_* reporter strain harboring a negative control plasmid or a CRISPRi plasmid against *cenI* was grown overnight in TY-Thi, subcultured 1:100 into TY-Thi with 1% xylose, grown to an OD_600_ of 0.5, and luciferase activity measured. When *cenI* was silenced, luciferase activity increased about 100-fold. Data are presented as the mean and SD from two experiments performed on different days with two technical replicates each. Statistical analysis was by one-way ANOVA, *n* = 4 (****P ≤ 0.0001). Strains: P*_cdr_0474_-sluc*^opt^/Neg con (MK261), P*_cdr_0474_-sluc*^opt^/sgRNA-*cenI* (MK260), Δ*cdr_0474* P*_cdr_0474_-sluc*^opt^/Neg con (MK338), Δ*cdr_0474* P*_cdr_0474_-sluc*^opt^/CRISPRi-*cenI* (MK339).

Recall that *cdr_0474* is a gene of unknown function that according to RNA-seq was induced 500-fold upon CenIR depletion. To verify the large induction, we endeavored to build reporter plasmids by cloning the *cdr_0474* promoter region upstream of codon-optimized genes for mCherry red fluorescent protein or NanoLuc luciferase (13–15). Despite repeated attempts, we were unable to recover intact plasmids, which might be toxic to *E. coli* due to depletion of rare tRNAs when the reporter genes are expressed at high levels. Ultimately, we were able to construct a P*_cdr_0474_::sluc^opt^* fusion in Tn916 in *Bacillus subtilis*. The reporter transposon was transferred by conjugation into *C. difficile*, where it integrated at the intergenic region upstream of *cdr_0029* as revealed by whole-genome sequencing. CRISPRi silencing of *cenIR* induced the secreted NanoLuc reporter (14) about 100-fold (Fig. 4), confirming that the reporter strain responds as expected based on the RNAseq results. Induction of the reporter was not affected by the deletion of *cdr_0474* (Fig. 4).

**Fig 5:**
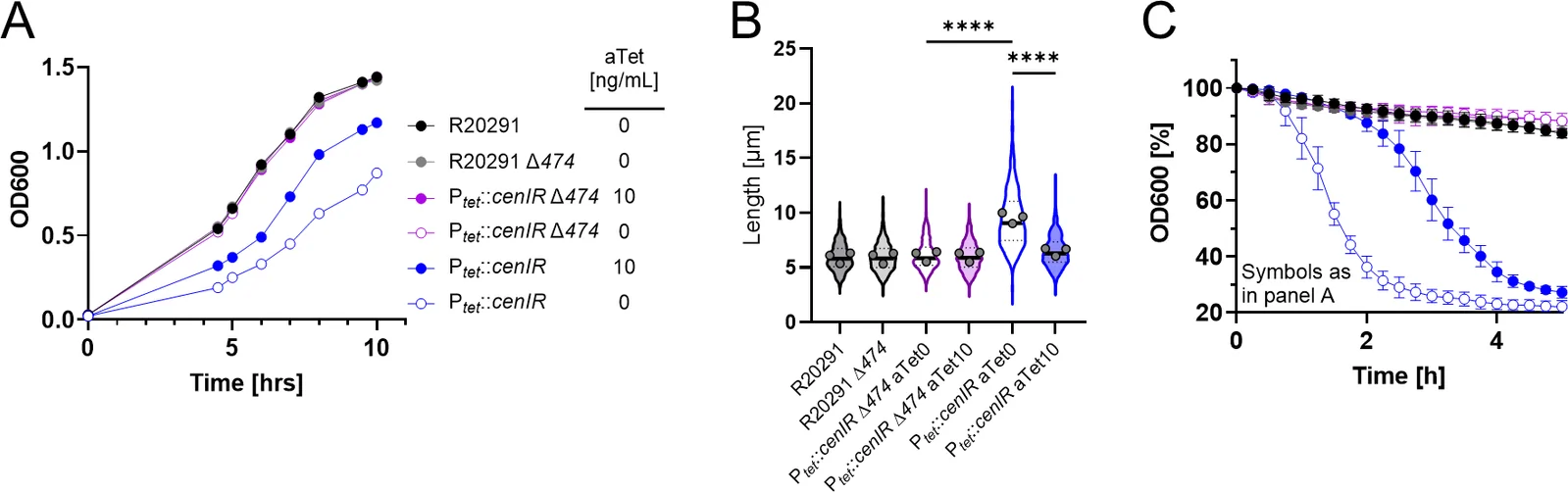
Deletion of *cdr_0474* rescues CenIR depletion phenotypes. (**A**) Growth curves. Strains grown overnight in TY (wild type) or TY with 2 ng/mL aTet (P_tet_::*cenIR*) were subcultured 1:50 into TY (wild type) or TY ± 10 ng/mL aTet (P_tet_::*cenIR*) and growth was monitored for 11 h. At 7.5 h, cultures were sampled for microscopy to determine cell length (**B**). Faster growing strains were back diluted at 6 and 9.5 h to ensure that all strains were at an OD_600_ ~ 0.65 when harvested for the lysis assay (**C**). Growth curves are representative of three experiments. Cell lengths are graphed as violin plots with pooled data from three experiments (≥200 cells each). Bars and dotted lines are the means and quartiles, respectively. Circles are the means of each experiment. Statistical analysis was by one-way ANOVA, *n* = 3 (****P ≤ 0.0001). Lysis is graphed as the mean ± SD of three experiments. Strains: R20291, R20291 Δ*cdr_0474* (MK252), P*_tet_::cenIR* (UM1364), P*_tet_::cenIR* Δ*cdr_0474* (MK254).

We next tested whether CDR_0474 contributes to any of the CenIR-depletion phenotypes. We started by introducing plasmids for CRISPRI knockdown of *cdr_0474* into the P*_tet_::cenIR* depletion background. Strikingly, knockdown of *cdr_0474* by CRISPRi with either of two different sgRNAs improved growth rate, reduced cell length and slowed lysis as compared to a control CRISPRi plasmid with a non-targeting sgRNA (Fig. 3; Fig. S1B). The reversal of CenIR depletion phenotypes upon CRISPRi silencing *cdr_0474* led us to investigate the effect of deleting *cdr_0474*. Indeed, a *cdr_0474* deletion in the depletion background completely restored wild-type growth, cell length, and lysis activity, while deletion of *cdr_0474* in the wild-type background had no effect (Fig. 5; Fig. S1C). The mild increase of Ldt1 seen by Western blot upon CRISPRi silencing *cenIR* was also abolished in the *Δcdr_0474* mutant (Fig. S4). Finally, we found that *cenIR* could be deleted in the Δ*cdr_0474* background. Both growth and lysis phenocopy the wild-type strain (Fig. S5A and B). Thus, the apparent lethality of deleting *cenIR* in an otherwise wild-type background is likely related to run-away expression of *cdr_0474*.

### Cwp6 is responsible for the lysis phenotype

**Fig 6:**
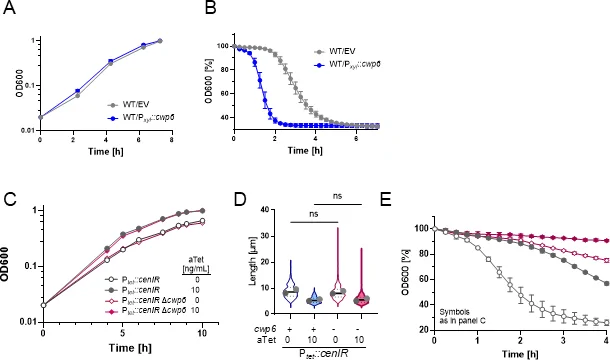
Cwp6 affects lysis but not growth rate or cell length. (**A and B**) Effect of *cwp6* induction on growth and lysis. Wild-type harboring empty vector (EV) or a P*_xyl_::cwp6* expression plasmid were grown in TY-thi with 1% xylose. At 7 h, samples were harvested for the lysis assay. The growth curves are representative of three experiments. Lysis is graphed as the mean ± SD of three experiments. (**C–E**) Effect of deleting *cwp6* on growth, cell length, and lysis. Strains grown overnight in TY with 2 ng/mL aTet were subcultured 1:50 into TY with 0 or 10 ng/mL aTet. Samples were harvested at 6.5 h for phase contrast microscopy to determine cell length. At 6 h, the 10 ng/mL aTet culture was back diluted ~5-fold so that all strains had an OD_600_ = ~0.7 at 10 h when cells were harvested for the lysis assay. The growth curves are representative of two experiments. Cell lengths are graphed as violin plots with data pooled from two experiments (≥200 cells each). Bars and dotted lines show the means and quartiles, respectively. Circles show the means of each experiment. The indicated differences are not statistically significant (one-way ANOVA, *n* = 2). Lysis is graphed as the mean ± SD of the two experiments. Strains: WT/EV (UM1173), WT/P*_xyl_::cwp6* (UM1352), P*_tet_::cenIR* (UM1364), P*_tet_::cenIR* Δ*cwp6* (MK237).

Elevated production of Cwp6, an annotated PG hydrolase, seemed likely to underlie the propensity for lysis when *C. difficile* is depleted of CenIR. To test this hypothesis, we overexpressed *cwp6* from a P*_xyl_* plasmid in the wild-type background. Although growth rate was not affected, cells lysed markedly faster in the Triton X-100 based lysis assay (Fig. 6A and B; Fig. S1D). Conversely, deleting *cwp6* in the P*_tet_::cenIR* depletion background strongly ameliorated the lysis phenotype but had little or no effect on growth or cell length (Fig. 6C and D; Fig. S1E). Similar results were obtained using CRISPRi to knock down expression of *cwp6* in the P*_tet_::cenIR* depletion background (Fig. 3). Based on these results, we attempted to delete *cenIR* in the Δ*cwp6* background using the same sgRNA and homology repair regions that failed to generate a *cenIR* deletion in the wild-type background. Deletions were easily obtained. The resulting Δ*cenIR* Δ*cwp6* mutant grew like wild-type, with no cell elongation or evidence of lysis (Fig. S5C and D). These results are consistent with Cwp6 causing the increased lysis seen upon depletion of *cenIR*. Moreover, the essentiality of *cenIR* is due to the lysis phenotype, not slower growth or elongation.

### Unsuccessful efforts to identify conditions that induce the *cenIR* regulon

We used our chromosomal P*_cdr_0474_::sluc^opt^* fusion to screen for conditions or signals that activate the regulon. Antibiotics were tested by growing the reporter strain in TY broth with a range of antibiotic concentrations. The antibiotics tested included two β-lactams (cefalexin, cefoperazone), additional cell wall-targeting drugs (bacitracin, ramoplanin, ristocetin), and a DNA gyrase inhibitor (novobiocin). A strain harboring the CRISPRi-*cenI* plasmid served as a positive control and showed 100-fold induction of luciferase activity in the presence of 1% xylose. None of the antibiotics affected luciferase even 1.5-fold. The lack of response to β-lactams is not surprising since CenR lacks a β-lactam-binding domain at the C-terminus, but we still considered it possible that CenIR might respond to cell envelope damage induced by β-lactams or other cell wall-targeting drugs. Either this is not the case, or we have yet to test the right compounds. Extending our speculation that the CenIR system might respond to perturbations in the cell wall, we assayed P*_cdr_0474_::sluc^opt^* expression after CRISPRi silencing a dozen cell envelope-associated genes: *pbp1, pbp2, fabK, murJ2, zapA, ddl, ispG, dxr, pgm2, murB, slpA,* and *walA*. The CRISPRi plasmids targeting these genes have been validated previously based on drastic loss of viability and/or cell morphology when knockdown is induced (8). Strains harboring these CRISPRi plasmids were subcultured into TY-Thi with 1% xylose and sampled after 5.5 h. The positive control, CRISPRi*-cenI* induced the reporter about 100-fold, while none of the other constructs increased luciferase activity even 1.5-fold.

### Deletion of *cenIR* does not affect the MIC of select antibiotics

Once it became possible to delete *cenIR* (by first deleting *cdr_0474* or *cwp6*), we could ask whether this regulator is important for responding to antibiotics. To this end, we used minimum inhibitory concentration (MIC) assays to compare wild-type R20291, Δ*cdr_0474,* and the Δ*cdr_0474* Δ*cenIR* double mutant. We tested three β-lactam antibiotics (ampicillin, methicillin, meropenem), two cell wall-acting antibiotics from different classes (bacitracin, vancomycin) and a replication inhibitor (novobiocin). However, sensitivities to these antibiotics were the same for all three strains (Table S2).

### BlaR homologs without a predicted transpeptidase domain are widespread

At the outset of this study, we thought the absence of a β-lactam-binding TP domain in CenR made this sensor an unusual BlaR-like protein. But close inspection of the six other BlaR-like proteins in strain R20291 revealed that while all are predicted membrane proteins with an M56 peptidase domain (PF05569) only one of them has a TP domain (PF00905) (Table S3). That one is the previously characterized canonical BlaR protein CDR_0412 (4). The remaining six BlaR-like proteins, including CenR, have unique C-terminal domains that are much shorter than a TP domain and, in most cases, are predicted to be largely unstructured (Table S3). Two of these novel BlaR-like proteins are encoded in an operon with a corresponding *blaI*-like gene (*cdr_2276/2277* and *cdr_2279/2280*). Interestingly, the latter pair is upstream of CDR_2278, which has a PG binding domain typically found in PG hydrolases (PF01471), suggesting that it might play a role in regulating cell wall homeostasis.

**Fig 7:**
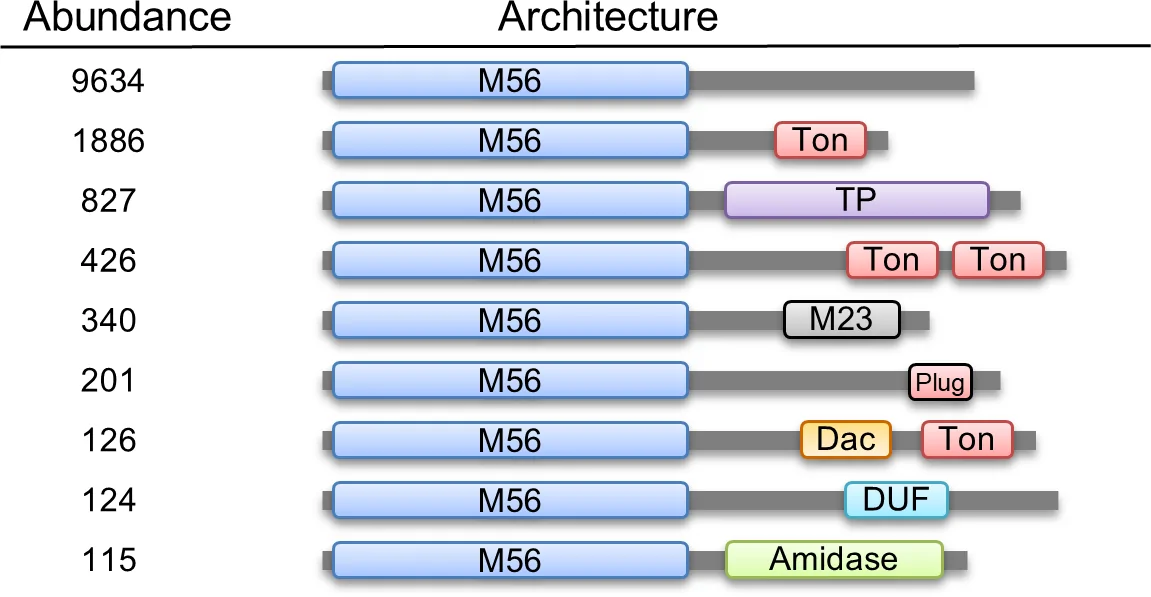
M56 domain proteins have diverse C-terminal domains. The M56 domain (PF05569) was used to search the InterPro database for homologs. The roughly 15,000 hits grouped into 254 domain architectures. Architecture arrangements with 100 or more entries are shown. Abbreviations are as follows. Ton, TonB_C domain (PF03544); TP, transpeptidase domain (PF00905); M23, Peptidase M23 domain (PF01551); Plug, TonB dependent receptor plug domain (PF07715); Dac, CarbopepD_reg_2 domain (PF13715); DUF, Domain of unknown function 4825 (PF16107); Amidase, Amidase_3 domain (PF01520).

These observations made us wonder whether it might be common for a BlaR-like protein to sense something other than a β-lactam antibiotic. We identified one published example, a non-canonical BlaR homolog (i.e., without the β-lactam-binding TP domain) that has been studied in both *Mycobacterium tuberculosis* and in *Mycobacterium abscessus* (16, 17). In *M. abscessus,* it has been linked to energy metabolism rather than antibacterial resistance. To search for additional atypical BlaR proteins, we used the InterPro database to retrieve roughly 15,000 protein entries with an M56 domain (PF05569) and then grouped these by domain architecture (18, 19). There were 254 different domain arrangements of which 9 included 100 or more entries (Fig. 7). The most abundant architecture is for an M56 domain followed by C-terminal regions of various lengths but with no identifiable domain. CenR falls into this category. The second most common architecture has a C-terminal TonB_C domain. Bona fide BlaR proteins with a TP domain that can bind β-lactams came in third on the list and represented only ~6% of “BlaR-like” homologs.

To verify that the BlaR homologs included in this analysis are sensors involved in regulating gene expression, and not, for example, proteins that have an M56 peptidase domain for some other reason, we looked for a closely linked BlaI-like protein. This analysis was performed using the EFI—Genome Neighborhood Tool (20, 21). About 96% of the BlaR homologs were within 5 genes of a predicted BlaI-like transcriptional repressor. In most cases, the BlaI homolog was upstream of the BlaR homolog.

## DISCUSSION

Here, we have identified and characterized a BlaIR-like regulatory system composed of the predicted transcriptional repressor CenI and the predicted membrane metalloprotease CenR. Although our studies were confined to strain R20291, *cenIR* is conserved in all 17 *C. difficile* strains in BioCyc (version 29.6 released 26 February 2026) (22). Unlike canonical BlaIR-like systems, which sense β-lactams to induce expression of a β-lactamase, CenIR is not implicated in β-lactam resistance. This conclusion is supported by three observations. (i) Deletion of CenIR had no effect on β-lactam resistance. (ii) The CenIR regulon does not include any predicted β-lactamases. (iii) The extracellular sensing domain of CenR is not a penicillin-binding domain. Of note, *C. difficile* has a conventional BlaIR pair that mediates induction of a β-lactamase (4); this BlaIR pair is distinct from CenIR.

CenIR caught our attention because a large-scale transposon mutagenesis indicated it is essential for vegetative growth (5). Essentiality is interesting because it is unexpected. BlaIR systems typically mediate β-lactam resistance, so they are dispensable under standard growth conditions. Indeed, CenIR scored as non-essential in our own Tn-seq analysis of *C. difficile* (8). Nevertheless, we were unable to delete the locus. To gain insight into the importance and role of CenIR, we used two depletion strains, one based on CRISPRi, the other on replacing the native promoter with P*_tet_*. In both cases, depleting cells of CenIR resulted in slower growth, elongation, and eventual cell lysis. Nevertheless, depletion was not overtly lethal in spot titer assays.

It is unclear why the *cenIR* system appears to be essential by some approaches but not others. In principle, discrepancy between Tn-seq and gene deletion (or between two different Tn-seq screens) can be explained by the gradual disappearance of viable but slow-growing mutants from the pool of Tn insertion mutants. The discrepancy between our inability to delete *cenIR* and the viability of the depletion strains in spot-titer assays might be due to leaky expression in the depletion strains, but in that case using CRISPRi to (further) silence *cenIR* expression in the P_tet_::*cenIR* stain should reduce viability, something we have not observed.

RNA-seq suggests the CenIR regulon is small, about 12 genes. All of these genes were induced upon CenIR depletion, as expected if CenI is a repressor. Because the experimental design involved targeting *cenIR* with CRISPRi, it is not possible to say whether *cenIR* expression is autoregulated. The most interesting gene in the regulon is *cdr_0474*, which is located 215 bp downstream of *cenIR* and was induced up to 500-fold. CDR_0474 is a small exported protein (10) unique to *C. difficile*. Its function is unknown, and its sequence offers no clues as to what it might be doing. Baseline levels of sequence reads in RNA-seq for CDR_0474 indicate expression is high even before depletion of CenIR (Table S1). High basal expression suggests CDR_0474 plays a structural role rather than a catalytic one.

*cdr_0474* is not essential by transposon sequencing, and we had no difficulty deleting the gene. Importantly, deleting *cdr_0474* suppressed the morphological and lysis defects associated with CenIR depletion, and it was now possible to delete *cenIR*. The CenIR regulon also includes two enzymes involved in peptidoglycan biogenesis, *ldt1* and *cwp6*, which were upregulated in RNA-seq by six- and sevenfold, respectively. Ldt1 is a peptidoglycan crosslinking enzyme. Follow-up experiments with Ldt1 failed to link it to any morphological defects. Cwp6 is a peptidoglycan hydrolase. Further investigation of Cwp6 revealed it mediates the lysis phenotype, but not slower growth or cell elongation. Importantly, deleting *cwp6* rendered CenIR no longer essential. Thus, *cenIR* is an example of genes that are only essential in the context of other genes.

Based on these findings, we propose a model for the essentiality of CenIR. In this model, CenI represses *cdr_0474*, which is highly induced in the absence of CenI. Excess CDR_0474 protein disrupts normal envelope biogenesis, which leads to increased expression of the cell wall amidase *cwp6*. Lethality is due to Cwp6-mediated lysis. We attempted to test this hypothesis by overexpressing *cdr_0474* from a plasmid. However, we did not find any viability or morphology defects, likely because we were unable to achieve high enough levels of expression.

As an aside, we call attention to an unexpected feature of the lysis assay that may be of use to researchers who study cell envelope biogenesis in *C. difficile*. This assay was not very reproducible in our hands until we realized the rate of lysis is surprisingly sensitive to the optical density at which cells are harvested. Cells sampled at lower OD lysed much faster than cells sampled at higher OD (Fig. S2). The assay behaved well once we took care to harvest cells at the same OD, which often required back-dilution of faster-growing strains. Why the rate of lysis is growth phase-dependent is an interesting question. The major driver is unlikely to be Cwp6 because RNA-seq indicates its expression increases about fourfold across the growth curve, i.e., *C. difficile* becomes less prone to lysis as Cwp6 levels increase (23). The expression of *cenIR* and *cdr_0474* is not very growth phase-dependent (23).

The physiological role of CenIR remains an open question. The location of CenR in the membrane, the morphological defects observed upon CenIR depletion, and the genes in the CenIR regulon all point to a role in cell envelope biogenesis or homeostasis. Nevertheless, we were unable to identify any antibiotics or mutations that induced a P*_cdr_0474_::sluc^opt^* reporter, nor did deletion of CenIR in the *Δcdr_0474* background sensitize *C. difficile* to any of the antibiotics we tested. By analogy to canonical BlaIR pairs, whatever signal activates CenR does so by binding to its C-terminal tail. Prediction programs indicate this region (amino acids 334–460) is mostly unstructured, although a small globular domain comprising amino acids 417–459 is weakly predicted by AF3 (Fig. S6). Unstructured domains can serve as tethers, binding sites for proteins, and probes for gaps in the peptidoglycan wall (24–28).

In closing, we note that only one of *C. difficile’s* seven annotated BlaIR-like gene pairs has a BlaR protein with a C-terminal penicillin-binding domain (4). The putative sensing domains in the remaining six BlaR proteins are diverse in that they are largely unstructured and not homologous to one another. Importantly, *C. difficile* is not an outlier in this regard. Of the roughly 15,000 BlaIR systems identified in the InterPro database, only about 6% are predicted to bind a β-lactam. Putative signal-binding domains in the remaining BlaR-like proteins are for the most part diverse, and it is not possible to predict what ligands they bind. Thus, the name “Bla” reflects how these regulatory systems were discovered and first characterized rather than a conserved or even broadly representative function. What the various BlaIR-like systems have in common is a shared mechanism for transducing extracellular signals to changes in gene expression. This mechanism is based on regulated proteolysis of a transcriptional repressor protein by a cognate membrane-bound sensor protease.

## MATERIALS AND METHODS

### Strains, media, and growth conditions

Bacterial strains used in this study are listed in Table 2. *C. difficile* strains were derived from R20291 (29). *C. difficile* was routinely grown in tryptone-yeast extract (TY) medium, supplemented as needed with thiamphenicol at 10 μg/mL (TY-Thi). TY medium consisted of 3% tryptone, 2% yeast extract, and 2% agar (for plates). Brian heart infusion (BHI) media was prepared per manufacturer’s (DIFCO) instructions. BHIS media is BHI supplemented with 5 g/L yeast extract and 4 g/L glucose. *C. difficile* strains were maintained at 37°C in an anaerobic chamber (Coy Laboratory Products) in an atmosphere of 2% H_2_, 5% CO_2_, and 93% N_2_. *Escherichia coli* strains were grown in LB medium at 37°C with chloramphenicol at 10 μg/mL and/or ampicillin at 100 μg/mL as needed. LB medium contained 1% tryptone, 0.5% yeast extract, 0.5% NaCl, and 1.5% agar (for plates). OD_600_ measurements were made with the WPA Biowave CO8000 tube reader in the anaerobic chamber.

**TABLE 2:** Strains used in this study

Strain	Genotype	Plasmid feature	Source
R20291	Wild-type *C. difficile* strain from UK outbreak (ribotype 027)		
CDE5470	R20291 P*_cdr_0474_*::*sluc^opt^*		This study
KB852	R20291 Δ*cwp6*		This study
MK212	R20291 P*_tet_*::*cenIR*/pMK49	P*_xyl_*::*cenI*	This study
MK216	R20291 P*_tet_*::*cenIR*/pMK42	CRISPRi *cdr_0474-1*	This study
MK217	R20291 P*_tet_*::*cenIR*/pMK43	CRISPRi *cwp6-1*	This study
MK218	R20291 P*_tet_*::*cenIR*/pMK44	CRISPRi *cwp6-2*	This study
MK227	R20291 P*_tet_*::*cenIR*/pIA68	CRISPRi *ldt1-1*	This study
MK228	R20291 P*_tet_*::*cenIR*/pIA69	CRISPRi *ldt1-2*	This study
MK229	R20291 P*_tet_*::*cenIR*/pIA102	CRISPRi *cdr_0474-2*	This study
MK230	R20291 P*_tet_*::*cenIR*/pBZ101	P*_xyl_* negative control	This study
MK231	R20291 P*_tet_*::*cenIR*/pIA34	CRISPRi negative control	This study
MK237	R20291 P*_tet_*::*cenIR* Δ*cwp6*		This study
MK252	R20291 Δ*cdr_0474*		This study
MK254	R20291 P*_tet_*::*cenIR* Δ*cdr_0474*		This study
MK260	R20291 P*_cdr_0474_*::sluc^opt^/pIA59	CRISPRi *cenI*	This study
MK261	R20291 P*_cdr_0474_*::sluc^opt^/pIA34	CRISPRi negative control	This study
MK330	R20291 Δ*cdr_0474*/pIA34	CRISPRi negative control	This study
MK331	R20291 Δ*cdr_0474*/pIA59	CRISPRi *cenI*	This study
MK333	R20291 Δ*cenIR Δcdr_0474*		This study
MK338	R20291 Δ*cdr_0474* P*_cdr_0474_*::sluc^opt^/pIA59	CRISPRi *cenI*	This study
MK339	R20291 Δ*cdr_0474* P*_cdr_0474_*::sluc^opt^/pIA34	CRISPRi negative control	This study
MK342	R20291 Δ*cwp6* Δ*cenIR*		This study
UM1173	R20291/pBZ101	P*_xyl_* negative control	This study
UM1352	R20291/pIA141	P*_xyl_*::*cwp6*	This study
UM1364	R20291 P*_tet_*::*cenIR*		This study
UM359	R20291/pIA34	CRISPRi negative control	(7)
UM469	R20291/pIA59	CRISPRi *cenI*	This study

### Plasmid and strain construction

Plasmids are listed in Table S4 and were constructed with HiFi DNA Assembly from New England Biolabs (Ipswich, MA). Oligonucleotide primers (Table S5) were synthesized by Integrated DNA Technologies (Coralville, IA). Regions that were constructed by PCR were verified by DNA sequencing. Plasmids were propagated in *E. coli* HB101/pRK24 and conjugated into *C. difficile* R20291 according to reference 9. Details relevant to plasmid construction are provided in Table S4.

### Strain construction by CRISPR editing

Gene deletions were achieved by CRISPR editing, using pIA123 as the parental vector (Addgene #234038). This plasmid carries a xylose-inducible *cas9* gene, an easily swapable sgRNA (MscI-NotI sites) under control of a constitutive promoter, as well as a unique PstI site for insertion of the homology repair regions (typically 500 bp upstream and 500 bp downstream of the desired deletion). We generally added the homology region first, followed by insertion of the sgRNA. We typically screened 3–5 sgRNA candidates for optimal killing upon induction of *cas9*. Constructs with different guides were conjugated into the appropriate *C. difficile* strain and grown overnight in TY-Thi. The overnight culture was serially diluted (10-fold dilutions), spotted on both TY-Thi and TY-Thi 1% xylose plates and growth evaluated after 17 h incubation at 37°C. A suitable guide will achieve about 10^4^-fold loss of viability upon plating on xylose. Strains harboring an editing plasmid with an optimal sgRNA were then plated for selection by spreading 100 µL of a diluted overnight culture (10^−1^ and 10^−2^ dilution) on TY-Thi 1% xylose. After overnight growth, ~8 colonies were restruck on TY 1% xylose. The next day, colony PCR was used to evaluate which candidates have the intended deletion. Successful candidates were then restruck for multiple rounds on TY to achieve plasmid loss, as evaluated by patching on TY vs TY-Thi plates. CRISPR editing was also used to insert the P*_tet_* promoter elements upstream of the *cenIR* operon. In that case, the sequence for *tetR* and regulatory elements was included between the homology regions.

### Colony PCR

To prepare template DNA for PCR, a single colony is picked into 50 µL ThermoPol reaction buffer (NEB) with 0.5 µL thermolabile ProteinaseK (NEB), incubated at 37°C for 30 min, and then the proteinase is heat deactivated for 10 min at 55°C. Of this, 1 µL is used as template in subsequent PCR.

### Construction of a P*_cdr_0474_-sluc^opt^* reporter strain using a modified Tn916 transposon

Because the *cdr_0474* promoter was toxic in *E. coli*, a reporter fusion was constructed in a Tn916 transposon directly in *Bacillus subtilis* BS49 (30). The reporter construct placed 300 nucleotides upstream of *cdr_0474* in front of the gene for secreted NanoLuc, *sluc*^opt^ (14). Five DNA fragments were amplified by PCR using the primers listed in Table S5. These fragments were (i) Tn916 Frag1 (primers 7889/7890; template pSMB47 [31]), (ii) the P*_cdr_0474_* promoter region (primers 7891/7892, template R20291 genomic DNA), (iii) the *sluc^opt^* reporter gene (primers 7893/7894; template pAP24 [14]), (iv) a spectinomycin resistance cassette (primers 7895/7896; template pDG1730 [32]), and (v) Tn916 Frag2 (primers 7897/7898; template pSMB47). The purified PCR products were combined by isothermal assembly, and the resulting construct was directly transformed into competent *B. subtilis* BS49. Transformants were selected on agar medium containing spectinomycin at 100 µg/mL. To confirm correct assembly and insertion of the reporter cassette into the Tn*916* element, it was amplified by colony PCR, and the PCR products were analyzed by Sanger sequencing. A confirmed Tn916 reporter construct was transferred from *B. subtilis* to *C. difficile* R20291 by conjugations as previously described (33). Transconjugants were selected on BHIS plates containing tetracycline (10 µg/mL), cefoxitin (8 µg/mL), and kanamycin (50 µg/mL). The presence of the P*_cdr_0474_-sluc^opt^* reporter in the final strain, CDE5470, was confirmed by PCR. Whole-genome sequencing of the final reporter strain determined the insertion to be in the intergenic region between *acoL* and *cdr20291_0029* at position 63422–63425.

### Microscopy

Cells were immobilized using thin agarose pads (1%) as described (15). Phase-contrast micrographs were recorded on an Olympus BX60 microscope equipped with a 100 × UPlanXApo objective (numerical aperture, 1.45). Micrographs were captured with a Hamamatsu Orca Flash 4.0 V2 + complementary metal oxide semiconductor (CMOS) camera. The image analysis tool MicrobeJ (34) was used to measure cell length. A minimum of 200 cells were measured for each condition.

### Live cell microscopy

Growing cells were imaged anaerobically according to reference 35 with the following modifications. Gene frames (size 125 µL from Thermo Scientific) attached to a microscope slide, 2% molten agarose (RPI), and 2X TY were brought into the anaerobic chamber the day before use, so they had time to become anaerobic. The agarose was kept at 68°C. Shortly before assembling the slides, the 2X TY was also warmed to 68°C and then mixed 1:1 with the molten agarose, and FM4-64 was added to a final concentration of 1 µg/mL. Five hundred microliters of this mixture was pipetted into the Gene frame well and covered with a second microscope slide. After about 5 min for the agarose to solidify, the microscope slide cover was removed, and bacterial samples were spotted on to the agarose pad. The slide was then sealed with a 24 × 50 mm cover slip and removed from the chamber. The samples were kept at 37°C and imaged every 5 min by phase contrast and red fluorescence microscopy with an inverted Nikon Ti2 Eclipse as described (36).

### Lysis assay

Lysis activity was determined as reported (9). Briefly, cell cultures (1 mL) were removed from the anaerobic chamber, pelleted, and resuspended in 700 mL of 0.01% Triton X-100 in 50 mM NaPO4 buffer (pH 7.4). Of this, three 200-mL replicates were pipetted into wells of a clear, flat-bottom 96-well plate. The turbidity was measured at 600 nm every 15 min for 10 h at room temperature in a plate reader (Tecan Infinite M200 Pro). Note that lysis activity is growth phase dependent (Fig. S2), so cultures were sampled for the assay at the same OD_600_, typically 0.6–0.8.

### Western blot

Expression level of Ldt1 was quantitated by Western blotting as described (12). Details for culture growth are given in the legend of Fig. S4.

### Culture growth and RNA isolation for RNAseq

Two independent cultures each of R20291/pIA59 (CRISPRi-*cenIR*) and of the negative control strain R20291/pIA34 were grown overnight in TY-Thi 1% NaCl. The next day, these were subcultured 1:30 into TY-Thi 1% NaCl containing 1% xylose and grown for 13h. Cultures were kept in late log phase by monitoring the OD_600_ and twice back-diluting fivefold once they had reached an OD_600_ of 0.5 (Fig. S3). Cells were fixed by rapidly mixing 25 mL culture with 25 mL ice-cold 1:1 ethanol-acetone that had been brought into the anaerobic chamber on dry ice. Fixed cells were stored at −80°C until further workup. RNA was isolated according to reference 9.

### Transcript profiling (RNAseq)

RNA samples were submitted to the Microbial Genome Sequencing Center (MiGS) in Pittsburgh, PA. MiGS performed Illumina stranded RNA library preparation paired with RiboZero Plus (per the manufacturer’s specifications) and sequenced on a NextSeq 500 using a 75cyc high-output flow cell. Fastq files were trimmed and filtered using a combination of Trimmomatic (37) and FastQC (38). Alignment, normalization, and differential expression were analyzed with LaserGene v18.1.0.426. Annotation was imported from NCBI with the additional information obtained from proGenomes (39). All subsequent analysis was performed in Excel and GraphPad Prism 9.

### Luciferase assays

Luciferase activity was measured with Nano-Glo reagents from Promega (Madison, WI). Working stock of the assay reagent refers to a 1:50 dilution of the Nano-Glo substrate in assay buffer. Immediately before use, the working stock was further diluted 1:100 in water. Bacterial cultures (50 or 100 µL) were added to a white 96-well plate, mixed with an equal volume of the diluted Nano-Glo substrate, and allowed to sit 5 min in the dark at room temperature. Luminescence was measured in a plate reader (Tecan Infinite M200 Pro) and reported as relative light units (RLU) normalized to cell density.

### Minimum inhibitory concentration

Susceptibility to antibiotics was determined in biological duplicate on two separate days as described in reference 9.

### Bioinformatics

The InterPro database (February 2026) was searched for entries with an M56 domain (PF05569) and then grouped by domain architecture (18, 19). To determine whether BlaR homologs were found adjacent to BlaI homologs, the roughly 15,000 hits from InterPro were downloaded and analyzed with bioinformatics tools at EFI (Enzyme Function Initiative, version February 2026) (20, 21). First, a sequence similarity network (SSN) is created with the Enzyme Similarity Tool (EFI-EST) using the default settings and finalizing without alignment threshold or length restriction. The SSN is transferred to the EFI Gene Neighborhood Tool (GNT) with the Neighborhood size set to within five genes. The resulting PFAM family co-occurrence table was used to calculate the frequency of finding PF05569 within five genes of BlaI (PF03965).

## Data Availability

RNA-seq data were submitted to the NCBI GEO repository and assigned accession number GSE327362.

## SUPPLEMENTAL MATERIAL

Supplemental figures jb.00196-26-s0001.pdf Figures S1 to S6.

Table S1 jb.00196-26-s0002.xlsx RNA-seq.

Video S1 jb.00196-26-s0003.mp4 WT-phase contrast.

Video S2 jb.00196-26-s0004.mp4 WT-FM4-64.

Video S3 jb.00196-26-s0005.mp4 CenIR-phase contrast.

Video S4 jb.00196-26-s0006.mp4 CenIR-FM4-64.

ASM does not own the copyrights to Supplemental Material that may be linked to, or accessed through, an article. The authors have granted ASM a non-exclusive, world-wide license to publish the Supplemental Material files. Please contact the corresponding author directly for reuse.

## References

[1] Cole SA, Stahl TJ. 2015. Persistent and recurrent *Clostridium difficile* colitis. Clin Colon Rectal Surg 28:65–69. doi:10.1055/s-0035-1547333 26034401 PMC4442717

[2] Berumen Alvarez O, Purcell EB. 2023. Expanding our grasp of two-component signaling in *Clostridioides difficile*. J Bacteriol 205:e0018823. doi:10.1128/jb.00188-23 37728603 PMC10601699

[3] Peacock SJ, Paterson GK. 2015. Mechanisms of methicillin resistance in *Staphylococcus aureus*. Annu Rev Biochem 84:577–601. doi:10.1146/annurev-biochem-060614-034516 26034890

[4] Sandhu BK, Edwards AN, Anderson SE, Woods EC, McBride SM. 2019. Regulation and anaerobic function of the *Clostridioides difficile* β-lactamase. Antimicrob Agents Chemother 64:e01496-19. doi:10.1128/AAC.01496-19 31611350 PMC7187622

[5] Dembek M, Barquist L, Boinett CJ, Cain AK, Mayho M, Lawley TD, Fairweather NF, Fagan RP. 2015. High-throughput analysis of gene essentiality and sporulation in *Clostridium difficile*. mBio 6:e02383. doi:10.1128/mBio.02383-14 25714712 PMC4358009

[6] Fuchs M, Lamm-Schmidt V, Sulzer J, Ponath F, Jenniches L, Kirk JA, Fagan RP, Barquist L, Vogel J, Faber F. 2021. An RNA-centric global view of *Clostridioides difficile* reveals broad activity of Hfq in a clinically important gram-positive bacterium. Proc Natl Acad Sci USA 118:e2103579118. doi:10.1073/pnas.2103579118 34131082 PMC8237595

[7] Müh U, Pannullo AG, Weiss DS, Ellermeier CD. 2019. A xylose-inducible expression system and a CRISPR interference plasmid for targeted knockdown of gene expression in *Clostridioides difficile*. J Bacteriol 201:e00711-18. doi:10.1128/JB.00711-18 30745377 PMC6597395

[8] Alberts ME, Kurtz MP, Müh U, Bernardi JP, Bollinger KW, Dobrila HA, Duncan L, Laster HM, Orea AJ, Pannullo AG, et al.. 2025. Analysis of essential genes in *Clostridioides difficile* by CRISPRi and Tn-seq. J Bacteriol 207:e0022025. doi:10.1128/jb.00220-25 40919935 PMC12548448

[9] Müh U, Ellermeier CD, Weiss DS. 2022. The WalRK two-component system is essential for proper cell envelope biogenesis in *Clostridioides difficile*. J Bacteriol 204:e0012122. doi:10.1128/jb.00121-22 35575581 PMC9210968

[10] Cafardi V, Biagini M, Martinelli M, Leuzzi R, Rubino JT, Cantini F, Norais N, Scarselli M, Serruto D, Unnikrishnan M. 2013. Identification of a novel zinc metalloprotease through a global analysis of *Clostridium difficile* extracellular proteins. PLoS One 8:e81306. doi:10.1371/journal.pone.0081306 24303041 PMC3841139

[11] Girinathan BP, Ou J, Dupuy B, Govind R. 2018. Pleiotropic roles of *Clostridium difficile* sin locus. PLoS Pathog 14:e1006940. doi:10.1371/journal.ppat.1006940 29529083 PMC5864091

[12] Bollinger KW, Müh U, Ocius KL, Apostolos AJ, Pires MM, Helm RF, Popham DL, Weiss DS, Ellermeier CD. 2024. Identification of a family of peptidoglycan transpeptidases reveals that *Clostridioides difficile* requires noncanonical cross-links for viability. Proc Natl Acad Sci USA 121:e2408540121. doi:10.1073/pnas.2408540121 39150786 PMC11348318

[13] Ransom EM, Ellermeier CD, Weiss DS. 2015. Use of mCherry Red fluorescent protein for studies of protein localization and gene expression in *Clostridium difficile*. Appl Environ Microbiol 81:1652–1660. doi:10.1128/AEM.03446-14 25527559 PMC4325159

[14] Oliveira Paiva AM, Friggen AH, Hossein-Javaheri S, Smits WK. 2016. The signal sequence of the abundant extracellular metalloprotease PPEP-1 can be used to secrete synthetic reporter proteins in *Clostridium difficile*. ACS Synth Biol 5:1376–1382. doi:10.1021/acssynbio.6b00104 27333161

[15] Ransom EM, Weiss DS, Ellermeier CD. 2016. Use of mCherryOpt fluorescent protein in *Clostridium difficile*. Methods Mol Biol 1476:53–67. doi:10.1007/978-1-4939-6361-4_5 27507333

[16] Sala C, Haouz A, Saul FA, Miras I, Rosenkrands I, Alzari PM, Cole ST. 2009. Genome-wide regulon and crystal structure of BlaI (Rv1846c) from *Mycobacterium tuberculosis*. Mol Microbiol 71:1102–1116. doi:10.1111/j.1365-2958.2008.06583.x 19154333

[17] Bonefont LE, Davenport HC, Chaton CT, Korotkov KV, Rohde KH. 2024. Atypical *Mycobacterium abscessus* BlaRI ortholog mediates regulation of energy metabolism but not β-lactam resistance. Mol Microbiol 122:583–597. doi:10.1111/mmi.15314 39308125

[18] Blum M, Andreeva A, Florentino LC, Chuguransky SR, Grego T, Hobbs E, Pinto BL, Orr A, Paysan-Lafosse T, Ponamareva I, et al.. 2025. InterPro: the protein sequence classification resource in 2025. Nucleic Acids Res 53:D444–D456. doi:10.1093/nar/gkae1082 39565202 PMC11701551

[19] Paysan-Lafosse T, Andreeva A, Blum M, Chuguransky SR, Grego T, Pinto BL, Salazar GA, Bileschi ML, Llinares-López F, Meng-Papaxanthos L, et al.. 2025. The Pfam protein families database: embracing AI/ML. Nucleic Acids Res 53:D523–D534. doi:10.1093/nar/gkae997 39540428 PMC11701544

[20] Zallot R, Oberg N, Gerlt JA. 2019. The EFI web resource for genomic enzymology tools: leveraging protein, genome, and metagenome databases to discover novel enzymes and metabolic pathways. Biochemistry 58:4169–4182. doi:10.1021/acs.biochem.9b00735 31553576 PMC7057060

[21] Oberg N, Zallot R, Gerlt JA. 2023. EFI-EST, EFI-GNT, and EFI-CGFP: enzyme function initiative (EFI) web resource for genomic enzymology tools. J Mol Biol 435:168018. doi:10.1016/j.jmb.2023.168018 37356897 PMC10291204

[22] Karp PD, Billington R, Caspi R, Fulcher CA, Latendresse M, Kothari A, Keseler IM, Krummenacker M, Midford PE, Ong Q, et al.. 2019. The BioCyc collection of microbial genomes and metabolic pathways. Brief Bioinform 20:1085–1093. doi:10.1093/bib/bbx085 29447345 PMC6781571

[23] Gu H, Shi K, Liao Z, Qi H, Chen S, Wang H, Li S, Ma Y, Wang J. 2018. Time-resolved transcriptome analysis of *Clostridium difficile* R20291 response to cysteine. Microbiol Res 215:114–125. doi:10.1016/j.micres.2018.07.003 30172297

[24] Ohashi T, Hale CA, de Boer PAJ, Erickson HP. 2002. Structural evidence that the P/Q domain of ZipA is an unstructured, flexible tether between the membrane and the C-terminal FtsZ-binding domain. J Bacteriol 184:4313–4315. doi:10.1128/JB.184.15.4313-4315.2002 12107152 PMC135210

[25] Gardner K, Moore DA, Erickson HP. 2013. The C-terminal linker of *Escherichia coli* FtsZ functions as an intrinsically disordered peptide. Mol Microbiol 89:264–275. doi:10.1111/mmi.12279 23714328 PMC3725778

[26] van der Lee R, Buljan M, Lang B, Weatheritt RJ, Daughdrill GW, Dunker AK, Fuxreiter M, Gough J, Gsponer J, Jones DT, et al.. 2014. Classification of intrinsically disordered regions and proteins. Chem Rev 114:6589–6631. doi:10.1021/cr400525m 24773235 PMC4095912

[27] Holmes JA, Follett SE, Wang H, Meadows CP, Varga K, Bowman GR. 2016. *Caulobacter* PopZ forms an intrinsically disordered hub in organizing bacterial cell poles. Proc Natl Acad Sci USA 113:12490–12495. doi:10.1073/pnas.1602380113 27791060 PMC5098656

[28] Brunet YR, Habib C, Brogan AP, Artzi L, Rudner DZ. 2022. Intrinsically disordered protein regions are required for cell wall homeostasis in *Bacillus subtilis*. Genes Dev 36:970–984. doi:10.1101/gad.349895.122 36265902 PMC9732909

[29] Stabler RA, He M, Dawson L, Martin M, Valiente E, Corton C, Lawley TD, Sebaihia M, Quail MA, Rose G, et al.. 2009. Comparative genome and phenotypic analysis of *Clostridium difficile* 027 strains provides insight into the evolution of a hypervirulent bacterium. Genome Biol 10:R102. doi:10.1186/gb-2009-10-9-r102 19781061 PMC2768977

[30] Browne HP, Anvar SY, Frank J, Lawley TD, Roberts AP, Smits WK. 2015. Complete genome sequence of BS49 and draft genome sequence of BS34A, *Bacillus subtilis* strains carrying Tn*916*. FEMS Microbiol Lett 362:1–4. doi:10.1093/femsle/fnu050 25673660

[31] Edwards AN, Pascual RA, Childress KO, Nawrocki KL, Woods EC, McBride SM. 2015. An alkaline phosphatase reporter for use in *Clostridium difficile*. Anaerobe 32:98–104. doi:10.1016/j.anaerobe.2015.01.002 25576237 PMC4385412

[32] Guérout-Fleury AM, Frandsen N, Stragier P. 1996. Plasmids for ectopic integration in *Bacillus subtilis*. Gene 180:57–61. doi:10.1016/s0378-1119(96)00404-0 8973347

[33] Bouillaut L, McBride SM, Sorg JA. 2011. Genetic manipulation of *Clostridium difficile*. *In* Curr Protoc Microbiol. Vol. Chapter 9:Unit 9A.2. 10.1002/9780471729259.mc09a02s20 PMC3615975 21400677

[34] Ducret A, Quardokus EM, Brun YV. 2016. MicrobeJ, a tool for high throughput bacterial cell detection and quantitative analysis. Nat Microbiol 1:16077. doi:10.1038/nmicrobiol.2016.77 27572972 PMC5010025

[35] Ribis JW, Nieto C, DiBenedetto NV, Mehra A, Kuhn P, Dong Q, Nagawa I, El Meouche I, Aldridge BB, Dunlop MJ, et al.. 2025. Unique growth and morphology properties of Clade 5 *Clostridioides difficile* strains revealed by single-cell time-lapse microscopy. PLoS Pathog 21:e1013155. doi:10.1371/journal.ppat.1013155 40397889 PMC12140426

[36] Yarrington KD, Sánchez Peña A, Limoli DH. 2020. Kinetic visualization of single-cell interspecies bacterial interactions. J Vis Exp, no. 162. doi:10.3791/61376 32831301

[37] Bolger AM, Lohse M, Usadel B. 2014. Trimmomatic: a flexible trimmer for Illumina sequence data. Bioinformatics 30:2114–2120. doi:10.1093/bioinformatics/btu170 24695404 PMC4103590

[38] Andrews S. 2010. FastQC: a quality control tool for high throughput sequence data. Available from: https://www.bioinformatics.babraham.ac.uk/projects/fastqc

[39] Mende DR, Letunic I, Huerta-Cepas J, Li SS, Forslund K, Sunagawa S, Bork P. 2017. proGenomes: a resource for consistent functional and taxonomic annotations of prokaryotic genomes. Nucleic Acids Res 45:D529–D534. doi:10.1093/nar/gkw989 28053165 PMC5210662

