# Supplementary material for: CenIR, an essential BlaIR-family regulatory system in C. difficile
Source: J Bacteriol. 2026 Jul 14;208(8):e00196-26. doi: 10.1128/jb.00196-26 (PMC13492132; doi:10.1128/jb.00196-26)
Supplement: Supplementary file 1 [file jb.00196-26-s0001.pdf]

Supplemental information for

CenIR, an essential BlaIR-family regulatory system in *C. difficile*

Micaila P. Kurtz[1], Ute Müh[1], David S. Weiss[1,2]*, Craig D. Ellermeier[1,2]*

[1]Department of Microbiology and Immunology, Carver College of Medicine, University of Iowa, Iowa City, IA, USA

[2]Graduate Program in Genetics, University of Iowa, Iowa City, IA, USA

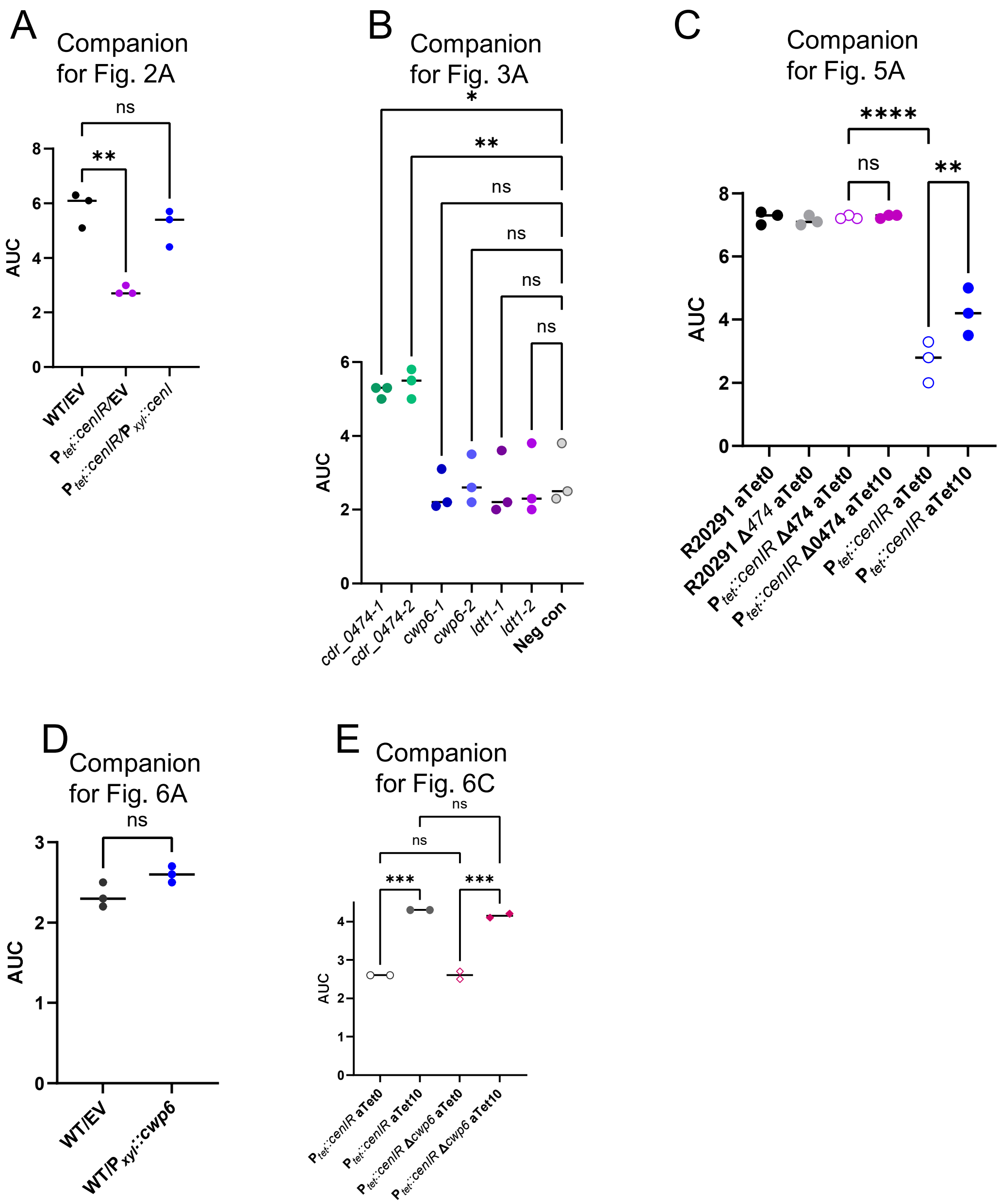

FIG S1. Statistical analysis of growth curves. Unpaired t-test if two conditions, one way ANOVA if more than two conditions. Color coding as in legend for original figures. Growth curves were compared based on area under the curve (AUC) rather than doubling time because there is no prolonged phase of the growth curve where doubling time is constant.

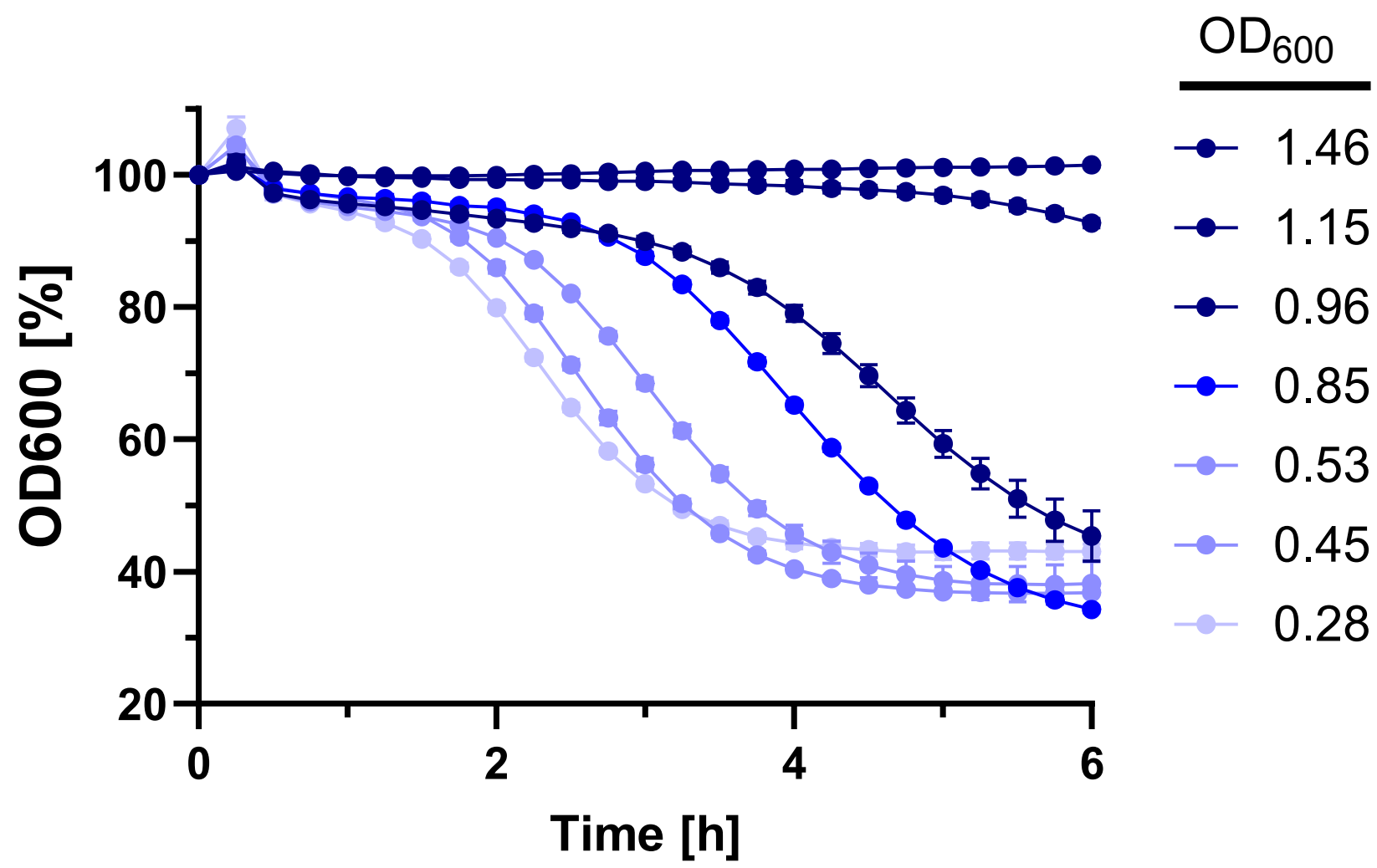

FIG S2. Lysis rate is dependent on OD_{600} at harvest. *C. difficile* R20291 was grown to different densities by generating a dilution series at subculture. All cultures were harvested at the same time for the lysis assay. Lysis is reported as turbidity clearance at 600 nm and graphed as percent of the starting turbidity. Data are representative of three independent experiments.

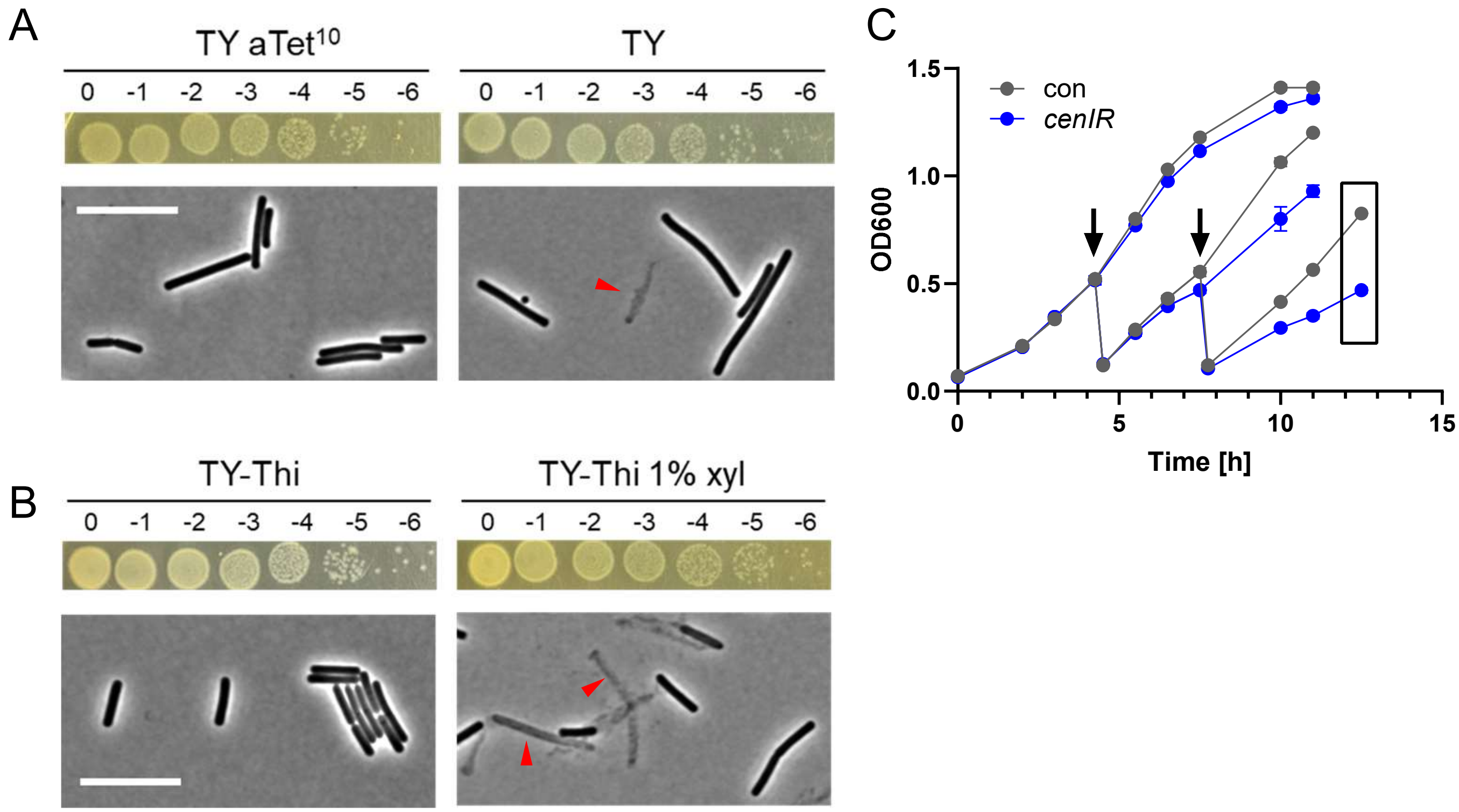

FIG S3. Effect of CenIR-depletion on viability. (A) Spot titer assay of the P_{tet}*::cenIR* depletion strain (UM1364). Serial dilutions of overnight cultures grown in TY with 2 ng/mL aTet were spotted on TY plates ±10 ng/mL aTet. After incubation at 37°C for ~18 h, plates were photographed and cells scraped from plates were imaged by phase contrast microscopy. Note elongation and lysis (red arrowhead) in the absence of aTet. (B) Spot titer assay of CRISPRi strain targeting *cenIR* (UM469). Serial dilutions of overnight cultures were spotted on TY-Thi plates ±1% xylose. After incubation at 37°C for ~18 h, plates were photographed and cells scraped from the plates and imaged by phase contrast microscopy. Note elongation and lysis (red arrows) in the presence of xylose. Spot titer assays are representative of two experiments. Size bars 10 µm. (C) Growth of CRISPRi-*cenIR* cultures for RNA-seq. Overnight cultures of R20291 harboring a negative control plasmid or a CRISPRi-*cenIR* plasmid were subcultured 1:30 into TY-Thi with 1% xylose, grown to an OD_{600} of 0.5 and back-diluted 1:50 twice (arrows). Cultures were harvested after 12.5 h (boxed). Data shown is representative of three experiments.

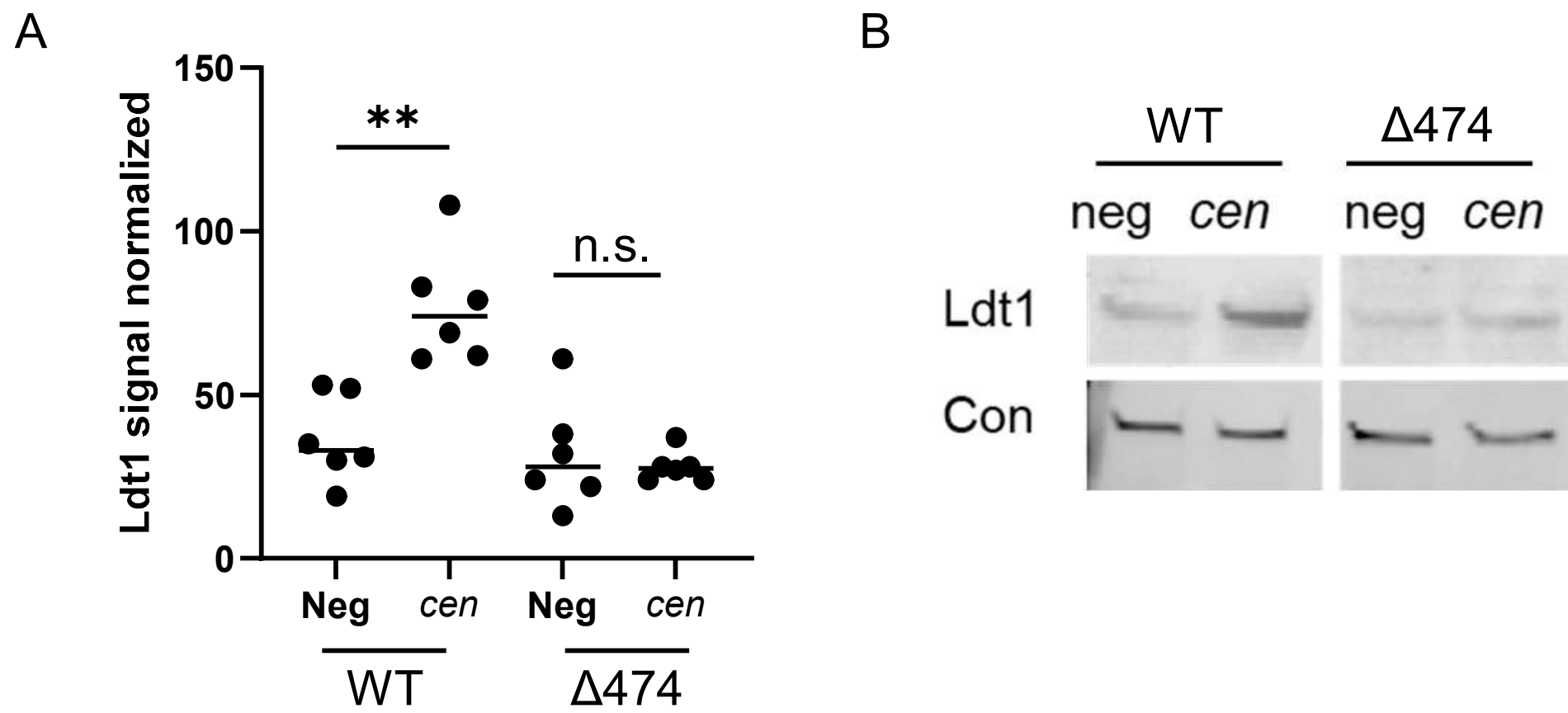

FIG S4. CenIR depletion increases Ldt1 abundance. (A) Quantification of Ldt1 based on Western blotting. Data are from three biological replicates, each with two technical replicates. Bars are the means. Statistical analysis was by Mann-Whitney t-test, n = 6 (** P<0.01). (B) Representative Western blots. R20291 or R20291 *Δcdr_0474* harboring a negative control plasmid (Neg) or a plasmid for CRISPRi knockdown of *cenIR* (*cen*) were grown in TY-Thi with 1% xylose to induce CRISPRi. Samples for Western blotting were taken at OD_{600} ~0.8. Primary antibody was anti-Ldt1 serum. Subsequent detection was with a secondary antibody conjugated to an IR dye. An unknown biotinylated protein detected with streptavidin IR probe served as a loading control (Con) and was used to normalize Ldt1 signal. Strains: WT/neg (UM359), WT/*cen* (UM469), Δ*cdr_0474*/neg (MK330), Δ*cdr_0474*/*cen* (MK331).

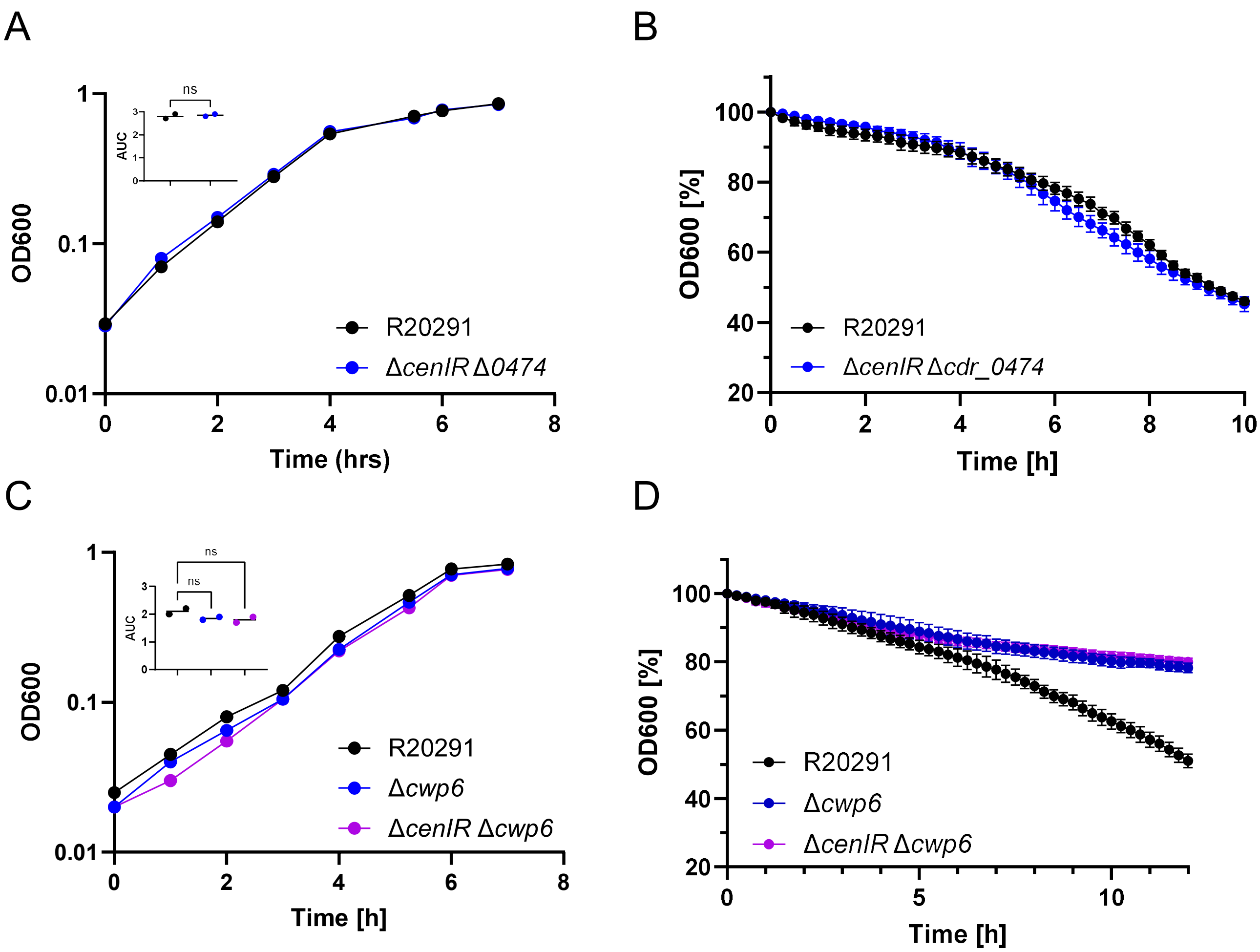

FIG S5. CenIR is not essential in the absence of CDR_0474 or Cwp6. The Δ*cenIR* Δ*cdr_0474* strain (A, B) and the Δ*cenIR* Δ*cwp6* strain (C,D) are do not have growth or lysis defects**.** Overnight-cultures were grown in TY were diluted 1:100 into TY and growth was monitored for 7 hours. Samples were harvest at OD_{600}~0.8 for the lysis assay. These experiments were done twice. Growth curves are representative. One way ANOVA analysis of replicates is shown in the insets. Lysis is graphed as the mean ± SD of the two experiments. Strains: R20291, Δ*cenIR* Δ*cdr_0474* (MK333), Δ*cwp6* (MK341), Δ*cenIR* Δ*cwp6* (MK342).

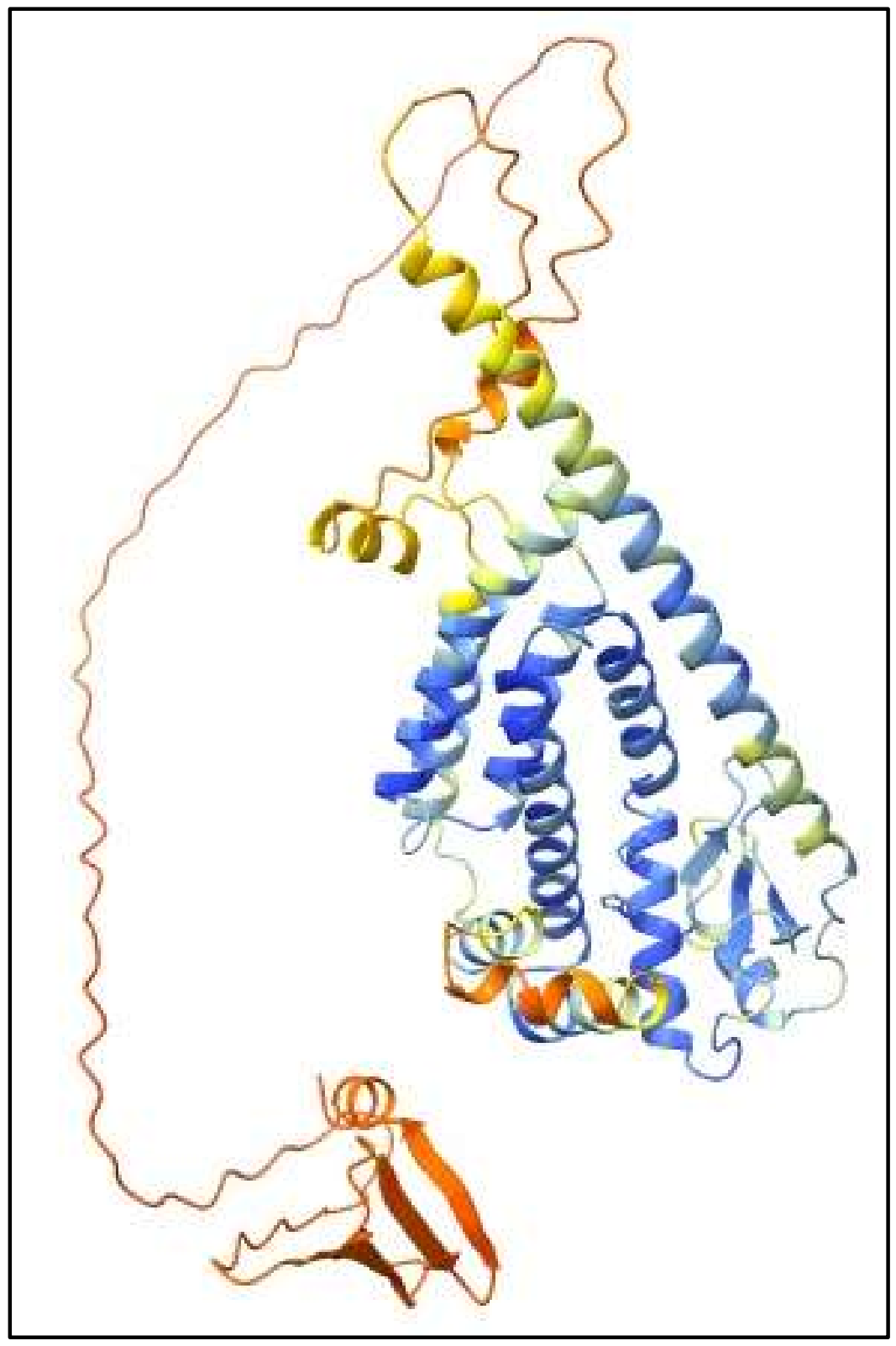

FIG S6. AF3 model of CenR. The M56 peptidase domain is mostly alpha helical and of relatively high confidence (blue) while the putative sensing domain is mostly unstructured and of low confidence (red). A small β-sheet is predicted with low confidence at the very C-terminus of the protein. Model confidence is based local distance difference test: Dark blue >90 (very high), light blue 89-70 (confident), yellow 69-50 (low), red <50 (very low).

VIDEO S1. Live cell microscopy. Phase contrast movie of wild type *C. difficile* R20291 grown on TY 1% agarose.

VIDEO S2. Live cell microscopy. Red fluorescence movie of wild type *C. difficile* R20291 grown on TY 1% agarose with membrane stain FM4-64.

VIDEO S3. Live cell microscopy. Phase contrast movie of the CenIR depletion strain (P_{tet}::*cenIR*) grown under depletion conditions (TY 1% agarose).

VIDEO S4. Live cell microscopy. Red fluorescence movie of the CenIR depletion strain (P_{tet}::*cenIR*) grown under depletion conditions (TY 1% agarose) with membrane stain FM4-64.

TABLE S2. Deletion of *cenIR* does not sensitize strains to select cell wall antibiotics

	Class	Target	MIC [μg/mL][1]		
			Wild type	Δ*cdr_0474*	Δ*cdr_0474* Δ*cenIR*
Ampicillin	β-Lactam	Cell wall	12.5	12.5	12.5
Methicillin	β-Lactam	Cell wall	12.5	12.5	12.5
Meropenem	β-Lactam	Cell wall	3.1	3.1	3.1
Bacitracin	Polypeptide	Cell wall	250-500	250-500	250-500
Vancomycin	Glycopeptide	Cell wall	0.4	0.4	0.4
Novobiocin	Coumarin	Replication	25	25	25

[1]Data averaged from two different days with two technical replicates each

TABLE S3. M56 domain proteins in *C. difficile* R20291

Locus	Gene	Length [AA][1]	Location of M56 domain	Number of C-terminal AA[2]	Annotated C-terminal domain[3]	Associated I protein
cdr_0182		377	9-276	101	none	no
cdr_0412	*blaR*	599	9-309	290	PF00905	*cdr_0413*
cdr_0473	*cenR*	460	4-296	164	none	*cdr_0472*
cdr_0531		337	9-272	65	none	no
cdr_2183		412	71-277	135	none	no
cdr_2276		340	8-277	63	none	*cdr_2277*
cdr_2279		344	12-281	63	none	*cdr_2280*

[1]Amino acids
[2]Amino acids after M56 domain
[3]None means no hits in HHPred search (1)

TABLE S4. Plasmid details

Plasmid	Relevant features	Parent vector	Restriction enzymes to digest parent vector	PCR primers	PCR template	Assembly	Comments	Reference
pAP114	P_{xyl}::*mChOpt catP*						Expression plasmid parent	2
pAP24	P_{tet}::*sluc*^{opt}						Template for *sluc*^{opt}	3
pBZ101	P_{xyl} empty vector *catP*						P_{xyl} negative control	4
pDG1730	Spectinomycin resistance *spc*						Template for *spc*	5
pIA102	P_{xyl}::*dCas9-opt* P_{gdh}::*sgRNA-cdr_0474 catP*	pIA33	SacI, BamHI	5641+4084	pIA33	HiFi	CRISPRi *cdr_0474* guide 1	this study
pIA123	P_{xyl} ::*cas9* P_{gdh} ::sgRNA-*cdr_985* catP						CRISPR editing plasmid (Addgene #234038)	6
pIA141	P_{xyl}::*cwp6 catP*	pAP114	SacI, BamHI	7252+7253	R20291	HiFi	Cwp6 expression	this study
pIA146	P_{xyl} ::*cas9* P_{gdh}::*sgRNA-cenI catP*	pIA123	MscI, NotI	7258+4084		HiFi	Cloning intermediate	this study
pIA148	P_{xyl}::*cas9* P_{gdh}::*sgRNA-cenI* , homology to insert P_{tet} in front of *cenIR catP*	pIA146	PstI	7261+7262<br>7267+7268<br>7265+7266	R20291<br>pRPF185<br>R20291	HiFi	CRISPR edit *cenIR* depletion strain	this study
pIA158	P_{xyl}::*cas9* P_{gdh}::*sgRNA-cdr_0474 catP*	pIA123	MscI, NotI	7681+4084	R20291	HiFi	Cloning intermediate	this study
pIA162	P_{xyl}::*cas9* P_{gdh}::*sgRNA-cdr_0474* , homology to delete *cenIR catP*	pIA158	PstI	7727+7728<br>7729+7730	R20291<br>R20291	HiFi	CRISPR edit *cdr_0474* deletion	this study
pIA167	P_{xyl}::*cas9* P_{gdh}::*sgRNA-cenI* , homology to delete *cenIR catP*	pIA146	PstI	7825+7826<br>7827+7828	R20291	HiFi	CRISPR edit *cenIR* deletion in wild type background	this study
pIA33	P_{xyl}::*dCas9-opt* P_{gdh}::*sgRNA-rfp catP*						Parent plasmid for CRISPRi constructs; Genbank MK368761, Addgene #120803	2
pIA34	P_{xyl}::*dCas9-opt* P_{gdh}::*sgRNA-neg catP*						CRISPRi neg con	2
pIA59/pCI4463	P_{xyl}::*dCas9-opt Pgdh::sgRNA-cenI catP*						CRISPRi *cenI*	7
pIA68	P_{xyl}::*dCas9-opt* P_{gdh}::*sgRNA-ldt1 catP*	pIA33	SacI, BamHI	4472+4084	pIA33	HiFi	CRISPRi *ldt1* guide 1	this study
pIA69	P_{xyl}::*dCas9-opt* P_{gdh}::*sgRNA-ldt1 catP*	pIA33	SacI, BamHI	4473+4084	pIA33	HiFi	CRISPRi *ldt1* guide 2	this study
pMK42	P_{xyl}::*dCas9-opt* P_{gdh}::*sgRNA-cdr_0474 catP*	pIA33	MscI, NotI	7349+4084	pIA33	HiFi	CRISPRi *cdr_0474* guide 2	this study
pMK43	P_{xyl}::*dCas9-opt* P_{gdh}::*sgRNA-cwp6 catP*	pIA33	MscI, NotI	7350+4084	pIA33	HiFi	CRISPRi *cwp6* guide 1	this study
pMK44	P_{xyl}::*dCas9-opt* P_{gdh}::*sgRNA-cwp6 catP*	pIA33	MscI, NotI	7351+4084	pIA33	HiFi	CRISPRi *cwp6* guide 2	this study
pMK49	P_{xyl}::*cenI catP*	pAP114	SacI, BamHI	7347+7348	R20291	HiFi	CenI expression	this study
pMK54	P_{xyl}::*cas9* P_{gdh}::*sgRNA-cenI* , homology to delete *cenIR catP*	pIA146	PstI	7825+8430<br>8431+7730	R20291<br>R20291	HiFi	CRISPR edit *cenIR* deletion in Δ*cdr_0474* background	this study
pPB001	P_{xyl}::*cas9* P_{gdh}::*sgRNA-pgdA* , homology to delete *cwp6 catP*	pIA123	PstI	7229+7230<br>7231+7232	R20291<br>R20291	HiFi	Cloning intermediate	this study
pPB103	P_{xyl}::*cas9* P_{gdh}::*sgRNA-cwp6* , homology to delete *cwp6 catP*	pPB001	MscI, NotI	7277+4237	pIA123	HiFi	CRISPR edit *cwp6* deletion	this study
pRPF185	P_{tet}::*gusA catP*						Template for P_{tet}	8
pSMB47	Tn*916*						Template for Tn*916*	9

TABLE S5. Oligonucleotides

Oligo	Sequence
4084	AACTTATAGGATCCGCGGCCGCTAGTCAGACATCATGCTGATCTAGA
4237	CTTATAGGATCCGCGGCCGCTAG
4463	AATTAAACTGTAAATGGCCA AAAGCTTTACAAACAAAATT GTTTTAGAGCTAGAAATAGC
4472	AATTAAACTGTAAATGGCCA TTCCTCTAAGTTTAAAGTAT GTTTTAGAGCTAGAAATAGC
4473	AATTAAACTGTAAATGGCCA TACTTAGGATAAGCACTCTT GTTTTAGAGCTAGAAATAGC
5641	AATTAAACTGTAAATGGCCATACCATCATTGCACTTATAA GTTTTAGAGCTAGAAATAGC
7229	CAGGAAGGGCGAATTCTGCACCCCAATAATAGCACTTGGAAGTAGCCC
7230	TTGCTTTAATATTTTTTATTATCCCCCCTAATTTTTTGGTTC
7231	ACCAAAAAATTAGGGGGGATAATAAAAAATATTAAAGCAAAACATATCATCTCCTG
7232	CCGGTCAGATCTGCACTGCAGCCCATTGTGCGCCAAATTT
7252	CGATAGTTATGAAGTGAGCTCAGATTTACATAATATTGAATTTATGTAATATAAAACAAG
7253	TTTAAAGTTTTATTAAAACTTATAGGATCCTTATTTTACTGTATCTTCTATTCCTATTCC
7258	AATTAAACTGTAAATGGCCATATTGTGATAACTATATTTGGTTTTAGAGCTAGAAATAGC
7261	CAGGAAGGGCGAATTCTGCAGCTAGTGGAGCAATTGAATATTTATCA
7262	TCTTATTTTTATGCTAGCTTGATTCTTATAAAAGACAGTTTTATCAATGTT
7265	AACAGATCTGAGCTCCTGCATATTTGAGGAGCGATTTTATATGGA
7266	CCGGTCAGATCTGCACTGCAGAATACAAATACCTTTTTAGGTAATTTTTTAAATAAAC
7267	AAACTGTCTTTTATAAGAATCAAGCTAGCATAAAAATAAGAAGC
7268	ATAAAATCGCTCCTCAAATATGCAGGAGCTCAGATCTG
7277	AATTAAACTGTAAATGGCCAGCGCCAGTAAAAAAACTTGC GTTTTAGAGCTAGAAATAGC
7347	CGATAGTTATGAAGTGAGCTCATAGTAATGACTATTGTGATAACTATATTTGA
7348	TTTAAAGTTTTATTAAAACTTATAGGATCCTCACTTTAATTTTTCAACAATTTGTTTG
7349	AATTAAACTGTAAATGGCCACATACTTTTCCAATTCAGTA GTTTTAGAGCTAGAAATAGC
7350	AATTAAACTGTAAATGGCCATCAGCCGCTAAAGCTACGTT GTTTTAGAGCTAGAAATAGC
7351	AATTAAACTGTAAATGGCCATAATTTAAAGTAGCTTCAAG GTTTTAGAGCTAGAAATAGC
7681	AATTAAACTGTAAATGGCCAGTATTTAATCATACTTACTT GTTTTAGAGCTAGAAATAGC
7727	CAGGAAGGGCGAATTCTGCAgATTTAAAAAATCAACTTATGTTACTTTTTTAGTT
7728	GCTTAATGTATTTAATCATACTTACTTAGATTTATGGTTACTCTATCCATGT
7729	TGGATAGAGTAACCATAAATCTAAGTAAGTATGATTAAATACATTAAGCTACT
7730	CCGGTCAGATCTGCACTGCAGAATAAATTTATAATCATATTATTACTCTATGTATTAAGC
7730	CCGGTCAGATCTGCACTGCAgAATAAATTTATAATCATATTATTACTCTATGTATTAAGC
7825	CAGGAAGGGCGAATTCTGCAGCTTGTTCTTATGTTACCGGTTTATA
7889	CTTGCGATTATACAGGTGGA
7890	CAAAGCGATTATCATTATAGATGATGAGTATCAACGTATTCCT
7891	AATACGTTGATACTCATCATCTATAATGATAATCGCTTTGTCATATC
7892	ATTTTCTCCTTTACTGCAGGTTATTTCATAACCAATCTTCCTTTCT
7893	GAAGATTGGTTATGAAATAACCTGCAGTAAAGGAGAAAATT
7894	ATTCTAGATTATATATGATACCTAGCGCCTACAGCCTTAT
7895	ATAAGGCTGTAGGCGCTAGGTATCATATATAATCTAGAATAAAATTAACTAAAATAATTATTAT
7896	GTTCAGATTCGGTAAAGTTCTGTTTCCACCATTTTTTCAATTT
7897	TTGAAAAAATGGTGGAAACAGAACTTTACCGAATCTGAACA
7898	GCTATAGTATAAGCCATACTTAAAACA
8430	AGTTTTTTATTATAGTAATGACAAGTAAGTATGATTAAATACATTAAGCTACT
8431	GTATTTAATCATACTTACTTGTCATTACTATAATAAAAAACTATCATAGTAGTC

References Supplemental Information

1. Zimmermann L, Stephens A, Nam SZ, Rau D, Kubler J, Lozajic M, Gabler F, Söding J, Lupas AN, Alva V. 2018. A Completely Reimplemented MPI Bioinformatics Toolkit with a New HHpred Server at its Core. J Mol Biol 430:2237–2243.
2. Müh U, Pannullo AG, Weiss DS, Ellermeier CD. 2019. A Xylose-Inducible Expression System and a CRISPR Interference Plasmid for Targeted Knockdown of Gene Expression in *Clostridioides difficile*. J Bacteriol 201:e00711–18.
3. Oliveira Paiva AM, Friggen AH, Hossein-Javaheri S, Smits WK. 2016. The Signal Sequence of the Abundant Extracellular Metalloprotease PPEP-1 Can Be Used to Secrete Synthetic Reporter Proteins in *Clostridium difficile*. ACS Synth Biol 5:1376–1382.
4. Müh U, Ellermeier CD, Weiss DS. 2022. The WalRK Two-Component System Is Essential for Proper Cell Envelope Biogenesis in *Clostridioides difficile*. J Bacteriol doi:10.1128/jb.00121-22:e0012122.
5. Guérout-Fleury AM, Frandsen N, Stragier P. 1996. Plasmids for ectopic integration in *Bacillus subtilis*. Gene 180:57–61.
6. Bollinger KW, Müh U, Ocius KL, Apostolos AJ, Pires MM, Helm RF, Popham DL, Weiss DS, Ellermeier CD. 2024. Identification of a family of peptidoglycan transpeptidases reveals that *Clostridioides difficile* requires noncanonical cross-links for viability. Proc Natl Acad Sci U S A 121:e2408540121.
7. Alberts ME, Kurtz MP, Müh U, Bernardi JP, Bollinger KW, Dobrila HA, Duncan L, Laster HM, Orea AJ, Pannullo AG, Rivera-Rosado JG, Torres FV, Ellermeier CD, Weiss DS. 2025. Analysis of essential genes in *Clostridioides difficile* by CRISPRi and Tn-seq. J Bacteriol 207:e0022025.
8. Fagan RP, Fairweather NF. 2011. *Clostridium difficile* has two parallel and essential Sec secretion systems. J Biol Chem 286:27483–93.
9. Edwards AN, Pascual RA, Childress KO, Nawrocki KL, Woods EC, McBride SM. 2015. An alkaline phosphatase reporter for use in *Clostridium difficile*. Anaerobe 32:98–104.
